# Investigating post-infection anxiety- and depression-like behaviors in a SARS-CoV-2 mouse model

**DOI:** 10.7150/thno.102752

**Published:** 2025-04-21

**Authors:** Qian Ge, Shan Zhou, Jose Porras, Panfeng Fu, Ting Wang, Jianyang Du, Kun Li

**Affiliations:** 1 Department of Anatomy and Neurobiology, University of Tennessee Health Science Center, Memphis, TN 38163, USA.; 2 Florida Research and Innovation Center, Cleveland Clinic, Port St. Lucie, FL 34987, USA.; 3 Center for Translational Science, Florida International University, Port St. Lucie, FL, 34987, USA.; 4 Neuroscience Institute, University of Tennessee Health Science Center, Memphis, TN, USA.

**Keywords:** SARS-CoV-2, Post-acute sequelae of COVID-19, Microglia, Amygdala, Anxiety- and depression-like behaviors.

## Abstract

**Rationale:** The COVID-19 pandemic, driven by SARS-CoV-2, has resulted in a wide range of neuropsychiatric symptoms associated with post-acute sequelae (PASC). However, the mechanisms by which SARS-CoV-2 impacts the brain and leads to persistent behavioral changes remain poorly understood. We hypothesize that SARS-CoV-2 exposure induces neuroinflammation and microglial activation, leading to anxiety- and depression-like behaviors in mice.

**Methods:** We established a SARS-CoV-2 mouse model using the virulent SARS2-N501Y_MA30_ strain to investigate its impact on the central nervous system (CNS). We assessed neuroinvasion via immunostaining of dsRNA and markers for neuronal, astrocyte, and microglia in brain slices. Behavioral changes were evaluated at 2 weeks, 2 months, and 4 months post-infection. Molecular and cellular analyses included bulk RNA-seq, Golgi-Cox staining, field excitatory postsynaptic potential (fEPSP) recordings, immunofluorescence, and quantitative real-time PCR (qRT-PCR) to assess gene expression, neuronal morphology, and microglial activation in the brain.

**Results**: We demonstrated that intranasal inoculation of SARS2-N501Y_MA30_ results in viral dissemination to multiple brain regions, including the amygdala and the prefrontal cortex (PFC). Behavioral assays indicated a marked elevation in anxiety- and depression-like behaviors post-infection. A comparative analysis of RNA expression profiles disclosed alterations in the post-infected brains. Additionally, we observed dendritic spine remodeling on neurons within the amygdala after infection. Infection with SARS2-N501Y_MA30_ was associated with microglial activation and a subsequent increase in microglia-dependent neuronal activity in the amygdala. Transcriptomic analysis of infected brains revealed the upregulation of inflammatory and cytokine-related pathways, implicating neuroinflammation in the pathogenesis of neuronal hyperactivity and behavioral abnormality.

**Conclusion:** Our findings provide evidence that SARS-CoV-2 neuroinvasion plays a critical role in the development of lasting behavioral sequelae observed in PASC. These data provide critical insights into the neurological consequences of SARS-CoV-2 infection and underscore microglia as a potential therapeutic target for ameliorating virus-induced neurobehavioral abnormalities.

## Introduction

As of March 3rd, 2025, COVID-19 has caused 777.5 million cases and 7.1 million deaths globally (WHO COVID-19 Dashboard). While vaccinations and treatments have reduced acute mortality, 10-20% of patients develop post-acute sequelae of SARS-CoV-2 (PASC, or "long COVID"), characterized by persistent symptoms lasting weeks to months 1. The abnormal PASC syndromes not only prolong COVID recovery and increase mortality but also aggravate the burden on the healthcare system 1. Neurological manifestations-cognitive deficits, autonomic dysfunction, fatigue, and mental health disturbances (such as anxiety and depression)- are particularly debilitating 2, 3. The mechanisms underlying SARS-CoV-2-induced neurological manifestations remain unclear, with proposed mechanisms including direct neuroinvasion, immune dysregulation, or both 4. SARS-CoV-2 RNA and proteins have been detected in autopsied brains, suggesting neurotropism via ACE2 receptor binding or tunneling nanotubes 5-7. Additionally, SARS-CoV-2 neuroinvasion can activate immune cells of the brain, potentially disrupting neural homeostasis and exacerbating neuropsychiatric symptoms 8.

As the brain's principal innate immune cells, glial cells - particularly microglia and astrocytes - play critical roles in neuroimmune function and disease. Microglia are highly dynamic 9-11, constantly surveying the microenvironment, monitoring neuronal activity 12, 13, and responding to infection and injury by releasing pro-inflammatory molecules and clearing apoptotic cells 14. Astrocytes, in turn, contribute to neural homeostasis by regulating synaptic transmission, maintaining the blood-brain barrier, and modulating neuroinflammation 15, 16. Under normal conditions, glial cells support neuronal function 17, 18; however, their hyperactivation can be pathogenic. Excessive microglial reactivity and astrocyte dysfunction have been linked to psychiatric symptoms such as anxiety and depression 19, 20, which have been widely reported during the COVID-19 pandemic. Despite the potential relationship between SARS-CoV-2 infection and neuroimmune dysregulation, the mechanisms by which the virus triggers pro-inflammatory microglial activation disrupts glia-neuron interactions, and alters neuronal activity remain inadequately studied.

Mouse models are crucial for studying PASC and its neurological effects. However, native mouse resistance to ancestral SARS-CoV-2 is due to low spike protein affinity for mouse ACE2 21, 22. While Ad5-hACE2 or AAV-hACE2 transduced and K18-hACE2 transgenic mice have been used to model acute SARS-CoV-2 infection and neurological consequences 23-25, they present limitations for long-term PASC studies. Ad5- or AAV-hACE2 intranasal transduction may restrict SARS-CoV-2 infection to the lungs, and K18-hACE2 can develop artificial brain infection 23-25. Therefore, to establish a more suitable in vivo model for studying the neurological sequelae of PASC, we infected mice with a novel mouse-adapted SARS-CoV-2 strain, SARS2-N501Y_MA30_. This highly virulent strain was isolated through serial passaging of a recombinant SARS-CoV-2/spike N501Y virus in Balb/c mice 26.

In SARS2-N501Y_MA30_-infected C57BL/6 mice, we detected viral titers, viral genomic RNA (gRNA), and subgenomic RNA (sgRNA) in the brain and lungs. Behavioral assays revealed persistent anxiety- and depression-like phenotypes at 14 and 60 days post-infection (open-field, elevated plus maze, tail suspension, and forced swim tests). Given the role of the amygdala in emotional regulation in rodents and humans 27, we identified viral RNA in neurons and microglial activation in this region. Additionally, exposure to SARS-CoV-2 spike protein enhanced microglia-dependent neuronal hyperactivity, suggesting a neuroinflammatory mechanism.

In summary, we hypothesized that SARS-CoV-2 exposure induces neuroinflammation and microglial activation, leading to anxiety- and depression-like behaviors in mice. The findings of this study provide valuable insights into the neurological consequences of SARS-CoV-2 infection and establish a unique mouse model of PASC for in vivo research. Understanding the neurological basis of COVID-19-related neuropsychiatric symptoms is essential for developing effective treatments to mitigate the long-term impact of PASC on mental health.

## Materials and methods

### Mice, viruses, and cells

Specific pathogen-free 6-9 weeks male and female Balb/c and C57BL/6 mice were purchased from the Jackson Laboratory. To ensure scientific rigor, experimental factors, such as sex, age, and weight are closely matched throughout the study. Experimental mice were maintained on a standard 12-hour light-dark cycle and received standard chow and water ad libitum. Animal care and procedures met the National Institutes of Health standards. All experimental protocols were approved by the Institutional Animal Care and Use Committees (IACUC) of the Cleveland Clinic-Florida Research and Innovation Center (CC-FRIC), protocol number 2797, and the University of Tennessee Health Science Center (UTHSC), protocol number 24-2696. The number of animals used in each experiment was determined based on power calculations to ensure statistical significance while adhering to the principles of reduction, refinement, and replacement. These calculations considered expected effect size, variability, and desired statistical power.

The mouse-adapted SARS-CoV-2-N501Y_MA30_ was provided by Drs. Stanley Perlman and Paul McCray at the University of Iowa, USA 26. The virus was propagated in Calu-3 cells and tittered by plaque assay in VeroE6 cells. Calu-3 cells were maintained in minimal essential medium (MEM) supplemented with 20% fetal bovine serum (FBS), 0.1 mM nonessential amino acids (NEAA), 1 mM sodium pyruvate, 2 mM l-glutamine, 1% penicillin and streptomycin, and 0.15% sodium bicarbonate (NaHCO_3_) at 37°C in 5% CO_2_. Vero E6 cells were grown in Dulbecco's modified Eagle medium (DMEM) supplemented with 10% FBS, 0.1 mM NEAA, 1 mM sodium pyruvate, 2 mM l-glutamine, 1% penicillin and streptomycin, and 0.15% NaHCO_3_ at 37°C in 5% CO_2_.

### Virus infection and titration

The research involving SARS-CoV-2 was conducted within the biosafety level 3 (BSL3) Laboratory at CC-FRIC. Balb/c or C57BL/6 mice were gently anesthetized using isoflurane and subsequently intranasally (i.n.) infected with 10^4^ PFU of SARS-CoV-2-N501Y_MA30_. Post-infection, daily monitoring and weight measurements of the mice were conducted. Tissues were aseptically collected and dissociated in PBS using disposable homogenizers. The viral preparation or supernatants from lung or brain tissue homogenates were subject to sequential dilution in DMEM. These diluted samples were then introduced to Vero E6 cells in 12-well plates to conduct plaque assays 28. After one hour of incubation, the viral inoculums were removed, and the cells were overlaid with a 1.2% agarose solution supplemented with 10% FBS. After 3-day incubation, the cells were fixed with formaldehyde, and the overlays were meticulously eliminated, facilitating visualization of the resulting plaques through the application of a 0.1% crystal violet stain.

### Behavioral experiments

C57BL/6 mice aged 6-9 weeks were utilized to investigate behavioral responses using a battery of tests as described in **Figure 2**. We conducted a well-established behavioral test battery in mice at indicated days after intranasal administration of SARS2-N501Y_MA30_ (10^4^ PFU/ mouse). For all behavior tests, mice were acclimated to the testing room for ≥ 30 minutes to minimize stress from novel environments. Mice are gently handled for 3-5 minutes/day for 3 days to reduce handling-related anxiety. See detailed descriptions below.

### Anxiety-like behaviors

#### Open field test (OFT)

Assessed exploratory and anxiety behaviors in an open field box for 6 minutes. The OFT was used to determine the general activity levels, gross locomotor activity, exploration habits of mice, and anxiety-like behaviors. The evaluation occurred within a cubic, white plastic enclosure measuring 30 cm × 30 cm × 30 cm. Mice were introduced to a corner of the arena and given 5 minutes to acclimate to the unfamiliar surroundings. Their activities were recorded via video. Subsequently, the recorded footage, encompassing metrics such as distance traversed, velocity, resting intervals, movement duration, and time spent in both the center and corners of the arena, was analyzed utilizing Anymaze software. This examination serves as an initial assessment of locomotor activity and anxiety-related behaviors, particularly focusing on the duration spent in the central area.

#### Elevated plus maze test (EPM)

The EPM is a gold-standard behavioral assay to evaluate anxiety-like behavior in rodents. It exploits the innate conflict between a mouse's curiosity to explore novel environments and its aversion to open, elevated spaces. Reduced time spent in open arms and fewer open-arm entries are interpreted as heightened anxiety. Evaluated anxiety-like behavior by recording entries into and time spent in the open arms of a maze for 6 minutes. It was conducted as previously described 29. The EPM apparatus, 40 cm high from the floor, consists of two open arms (35 × 10 cm) and two closed arms (35 × 10 cm), those two parts stretch perpendicular to each other and connect in a center platform (5 cm). Mice were placed in the center zone facing an open arm and allowed to explore the maze freely for 5 minutes. The time spent in the open arms was measured, and the percentage of open-arm time was calculated by dividing the time spent in the open arms by the total time spent in both the open and closed arms (5 minutes). Entries into the open arms were analyzed by calculating the percentage of open-arm entries relative to the total number of entries into both the open and closed arms.

### Depression-like behaviors

#### Tail suspension test (TST)

The TST is a widely used behavioral assay to evaluate depression-like states in rodents, particularly "behavioral despair" or learned helplessness. Mice suspended by their tails initially struggle to escape but eventually adopt a posture of immobility, which is interpreted as a resignation to stress. A sound-attenuated testing chamber (30 × 30 × 40 cm) constructed from opaque acrylic to prevent visual distractions. A horizontal bar or hook system is positioned 30-50 cm above the chamber floor, allowing mice to hang freely without touching surfaces. Adhesive tape is used to suspend mice by the tail, ensuring secure attachment while minimizing tissue damage. Mice are suspended by taping the tail 1-2 cm from the tip to prevent grip-based escape attempts. The tape is wrapped securely but not tightly to avoid ischemia. The trial lasts 6 minutes, with the first 1-2 minutes often excluded from analysis to account for initial vigorous struggling. Record the duration of immobility during the predetermined time period (6 minutes) and analyze utilizing Anymaze software. The state of immobility is defined by the absence of movement (less than 10% area change). The detection of immobility time in these tests serves as a behavioral measure to evaluate the impact of various interventions.

#### Forced swim test (FST)

The FST is used to assess depression-like behavior in rodents by measuring their response to an inescapable water environment. Increased immobility time is interpreted as behavioral despair, while decreased immobility suggests antidepressant-like effects. A transparent Plexiglas cylinder (height: 25-30 cm; diameter: 15-20 cm) filled with 18-20 cm of water (23-25°C) to prevent mice from touching the bottom or escaping. Water depth is standardized to ensure mice cannot stabilize by tail-touching. Place the mice individually in a water-filled container for 6 minutes and record their swim and immobility behaviors. The trial duration is 6 minutes, with the first 2 minutes excluded from the analysis to focus on stable behavioral responses, which were analyzed utilizing Anymaze software. Compile the data and calculate the mean immobility time for each experimental group.

### SARS-CoV-2 spike protein ELISA

C57BL/6 mice were intranasally infected with 10^4^ PFU of SARS-CoV-2-N501Y_MA30_, as previously described. Plasma was collected at 2, 4, 6, 8, and 14 days post-infection (dpi) from both infected and mock-infected mice using blood collection tubes (Becton Dickinson, Franklin Lakes, NJ, USA, catalog# 365985) via submandibular blood collection. Plasma SARS-CoV-2 spike protein levels were determined using a SARS-CoV-2 (2019-nCoV) Spike RBD ELISA Kit (Sino Biological Inc., Beijing, China, catalog# KIT40592) according to the manufacturer's instructions. Briefly, plasma samples were diluted according to the manufacturer's instructions. 100 μl of diluted standards or plasma samples were added to each well of the pre-coated plate. The plate was incubated for the time indicated in the manufacturer's protocol. Following this incubation, wells were washed three times with 300 μl of 1× wash buffer. 100 μl of HRP-conjugated secondary antibody was added to each well, and the plate was incubated for 1 hour at room temperature. After washing, 100 μl of substrate solution was added and incubated for 20 minutes at room temperature. The reaction was stopped by adding 100 μl of stop solution to each well. The absorbance was measured at 450 nm using a Synergy Neo2 hybrid multimode plate reader (BioTek, Winooski, VT, USA).

### Bulk RNA sequencing

C57BL/6 mice were intranasally infected with 10^4^ PFU of SARS-CoV-2-N501Y_MA30_, as described previously. At specified time points post-infection, whole brains were harvested for RNA extraction using the RNeasy Lipid Tissue Mini Kit (Qiagen, Hilden, Germany, catalog# 74804). RNA concentration and purity were determined by NanoDrop 2000 spectrophotometer (Thermo Fisher Scientific, Wilmington, DE, USA). Library preparation was performed with the Illumina Stranded mRNA Prep Kit (Illumina, San Diego, CA, USA, catalog# 20040532), adhering to the manufacturer's instructions. Sequencing was executed on the DNBSEQ-G400 platform (Innomics Inc. Cambridge, MA, USA). Data analysis and visualization were conducted on the Dr. Tom online analysis platform, utilizing the following pipeline: 1. Data Cleaning: Sequencing data was filtered with SOAPnuke, yielding clean reads stored in FASTQ format. 2. Alignment: Clean reads were aligned to the reference genome using HISAT2. 3. Fusion Gene Detection: Ericscript (0.5.5-5) was employed to identify fusion genes. 4. Differential Splicing Gene (DSG) Analysis: rMATS (v4.1.1) was utilized for detecting DSGs. 5. Gene Set Alignment: Bowtie2 aligned the clean reads to the gene set. 6. Expression Quantification: RSEM (v1.2.28) calculated gene expression levels, providing read count, FPKM, and TPM metrics.7. Differential Expression Gene (DEG) Analysis: DESeq2 conducted DEG analysis, with a significance threshold set at Q value ≤ 0.05. 8. Visualization: A heatmap of DEGs was generated using Pheatmap, based on the DEG analysis results. 9. Phenotypic Insight: GO (Gene Ontology, http://www.geneontology.org/) and KEGG (Kyoto Encyclopedia of Genes and Genomes, https://www.kegg.jp/) enrichment analyses of annotated DEGs were performed using Phyper, based on the Hypergeometric test. Significance levels of terms and pathways were adjusted by Q value, with a stringent cutoff (Q value ≤ 0.05). The bulk RNA-sequencing data generated in this study are publicly available in the Gene Expression Omnibus (GEO) under accession number GSE287550.

### Quantitative real-time PCR analysis of viral RNA

Total cellular RNA was isolated using the Direct-zol RNA miniprep kit (Zymo Research, Irvine, CA, catalog# R2063) following the manufacturer's protocol including a DNase treatment step. 200 ng of total RNA was used for first-strand cDNA synthesis with the High-Capacity cDNA Reverse Transcription Kit (Applied Biosystems, Waltham, MA, catalog# 4374967). The resulting cDNA was used to quantify the SARS-CoV-2 RNA levels by real-time quantitative PCR using SYBR Green Universal Master Mix (Applied Biosystems, Waltham, MA, catalog# 4309155). Average values from duplicates of each sample were used to calculate the viral RNA level relative to the GAPDH gene and presented as 2^-ΔCT, as indicated (where CT is the threshold cycle). CT values of gRNA and sgRNA from uninfected mice (0 dpi) are constantly > 35. The sequences of the primers used are listed in **Table S1**.

### Brain slice preparation, S1 protein perfusion, and fEPSP recordings

Mice were euthanized with overdosed isoflurane and whole brains were dissected into pre-oxygenated (5% CO_2_ and 95% O_2_) ice-cold high sucrose dissection solution containing (in mM): 205 sucrose, 5 KCl, 1.25 NaH_2_PO_4_, 5 MgSO_4_, 26 NaHCO_3_, 1 CaCl_2_, and 25 Glucose and sliced into 300 µm on a Leica VT1000S vibratome 30. The transverse amygdala slices were then transferred into the normal artificial cerebrospinal fluid (ACSF) containing (in mM): 115 NaCl, 2.5 KCl, 2 CaCl_2_, 1 MgCl_2_, 1.25 NaH_2_PO_4_, 11 glucose, 25 NaHCO_3_, bubbled with 95% O_2_/5% CO_2_, pH 7.35 at 20°C-22°C. Slices were incubated in the ACSF at least 1 hour before recording. Individual slices were transferred to a submersion-recording chamber and were continuously perfused with the 5% CO_2_/95% O_2_ solution (~3.0 ml/min) at room temperature (20°C-22°C). The Recombinant SARS-CoV-2 spike protein (B.1.351, Beta Variant) in its homotrimeric form, containing S1+S2 subunits and encompassing amino acids 16-1213 (BPSbIoscience, catalog# 510333, purity ≥ 90%, San Diego, CA, USA) was diluted to 167 ng/ml in ACSF 31 and perfused to the brain slices. We chose the B.1.351 spike variant due to its key mutations, particularly K417N, E484K, and N501Y, which are also found in SARS-CoV-2-N501Y_MA30_ (K417M, E484K, Q493R, Q498R, N501Y). For the fEPSP experiments, neurons were held in current-clamp mode with a pipette solution containing (in mM): 2 KCl (mOsm= 290, adjusted to pH 7.25 with KOH). A concentric bipolar stimulating electrode (FHC Inc., Bowdoin, ME) was positioned in the cortical inputs in the amygdala slices. Away from the stimulating electrode around 400 μm is a glass recording electrode. EPSPs were recorded in current-clamp mode every 20 seconds and continuously recorded the EPSPs for at least 1 hour. Data was acquired at 10 kHz using Multiclamp 700B and pClamp 10.

### ATP measurement

Brains were removed from the skull and dissected. Brain slices were then placed in 200 µl ice-cold PBS, in the presence of vehicle or spike protein (167 ng/ml) for the indicated time (10, 30, 60 minutes). To quantify ATP released from the brain slice, we employed an ATP Determination kit (Invitrogen, Waltham, MA, USA, catalog# A22066). Briefly, a 100 µl reaction mixture was added to the 96-well cell culture plate, which contains a 10 µl sample or standard solution. After 15 minutes of incubation in the dark, the plate was read using a Synergy Neo2 hybrid multimode microplate reader (BioTek, Winooski, VT, USA). ATP concentrations were determined by reference to a standard curve.

### Histology

Tissues (lungs, brain) were collected and fixed in zinc formalin. Following fixation, the lungs were processed for paraffin embedding and sliced into a 4 μm section and the brain was sectioned into 30 µm by a vibratome for subsequent hematoxylin and eosin (H&E) staining by Immunohistochemistry Core of Cleveland Clinic Lerner Research Institute and Immunohistochemistry Core at the University of Tennessee Health Science Center. We have used two serial lung sections (six fields/section) from each animal and a total of 4 to 5 animals per group. Acute lung injury severity was evaluated with ATS guidelines 32 for neutrophil infiltration in alveolar and interstitial space, hyaline membranes, alveolar wall thickening, and proteinaceous debris deposition. Briefly, a scoring system (0-2) was employed for each of the criteria mentioned. An average score of 0 indicated absence of injury, 1 indicated mild to moderate injury, and 2 indicated severe injury.

### Immunofluorescence and immunohistochemistry

Following the behavioral procedures indicated in the text and figures, the mice were euthanized with overdosed isoflurane and were fixed in Zinc Formalin. Following fixation, we used a vibratome (Leica VT-1000S) to dissect 30 µm amygdala coronal slices, which were collected in ice-cold PBS. To complete immunofluorescence staining, slices were placed in Superblock solution (Thermo Fisher Scientific) plus 0.2% Triton X-100 for 1 hour and incubated with primary antibodies (1:1000 dilution) at 4°C for 24 hours 30. Primary antibodies we used include mouse monoclonal antibody to dsRNA (Millipore catalog# MABE1134); Rabbit polyclonal to Iba1 (Abcam catalog# ab108539); Rabbit polyclonal to GFAP (Abcam catalog# ab7260) and mouse anti-NeuN (Cell Signaling catalog# 24307). We then washed and incubated slices for one hour with secondary antibodies diluted at a ratio of 1:200 (Alexa Fluor 488 goat anti-rabbit IgG (H+L) (Thermo Fisher Scientific catalog# A-11008); Alexa Fluor 594 goat anti-mouse IgG (H+L) (Thermo Fisher Scientific catalog# A-11032); Alexa Fluor 647 goat anti-mouse IgG (H+L) (Thermo Fisher Scientific catalog# A-21235). VectaShield H-1500 (Vector Laboratories catalog# H-1500) was used to mount slices, while regular fluorescent DIC microscopy and confocal microscopy were used to image the slices.

For SARS-CoV-2 antigen detection, tissue slides underwent a series of incubation steps. Initially, slides were treated with a blocking reagent (10% normal goat serum) for 30 minutes to reduce non-specific binding. Subsequently, a rabbit polyclonal antibody against the SARS-CoV-2 nucleocapsid protein (dilution: 1:4,000, catalog# 40143-T62, Sino Biological) was applied to the slides for 15 minutes. Following the primary antibody incubation, slides were further incubated with Rabbit Envision (Dako) and diaminobenzidine (Dako) as a chromogen to visualize the antigen-antibody complexes.

### Dendrites and spine morphologic analyses

After their behavioral testing, we measured the density of dendritic spines on amygdala neurons by using Golgi staining. Mice brains were collected, fixed, and processed for the Golgi staining according to the protocol provided by the FD Rapid Golgi Stain Kit (PK401, FD NeuroTechnologies). All images were deconvolved within the Zeiss Application Suite software. The number of dendritic spines was analyzed using plug-in SpineJ in image J as described 33, with modifications. Spines were examined on dendrites of amygdala neurons that satisfied the following criteria: (1) presence of untruncated dendrites, (2) dark and consistent Golgi staining throughout all dendrites, and (3) visual separability from neighboring neurons. We counted the number of dendritic spines along the second dendritic branch at distances from 50 to 100 µm from the soma, in images obtained at 630× magnification. For each neuron, 3-5 dendritic segments 10 µm in length were analyzed. For each group, 10-21 neurons from 4 mice were analyzed. The analysis of dendritic spines includes the number of spines and spine density, which are critical indicators of synaptic function 34, 35. Spines were classified as thin if they had a long, slender neck and small, Spine length > 0.5 µm; Head perimeter = 2 μm to 3 μm; mushroom, if they had a well-defined, thick neck and spine length > 0.5 µm; Head perimeter ≥ 3 µm; or stubby, if they were short and thick, without a distinguishable neck, spine length ≤ 0.5 µm; or filopodia if they were long and curved, spine length > 0.5 µm, head perimeter ≤ 2 μm.

### Statistical analysis

One-way or two-way analysis of variance (ANOVA) and Tukey's post hoc multiple comparison tests were used for the statistical comparison of multiple (≥3) groups. An unpaired Student's t-test was used to compare results between the two groups. p< 0.05 was considered statistically significant, and we did not exclude potential outliers from our data except the ones that met our exclusion criteria (refer to the individual Materials and Methods sections for details). The graphing and statistical analysis software GraphPad Prism 10 was used to analyze statistical data, which was presented as means ± SEM. Sample sizes (n) are indicated in the figure legends, and data are reported as biological replicates (data from different mice, different cells, or different brain slices). Each group contained tissues pooled from 4-5 mice. Due to variable behavior within groups, we used sample sizes of 8-22 mice per experimental group as we previously described in earlier experiments 30. In behavioral studies, animals were randomly assigned to experimental groups based solely on their cage numbers.

## Results

### Neuroinvasion following respiratory infection of SARS-CoV-2

**Figure 1:**
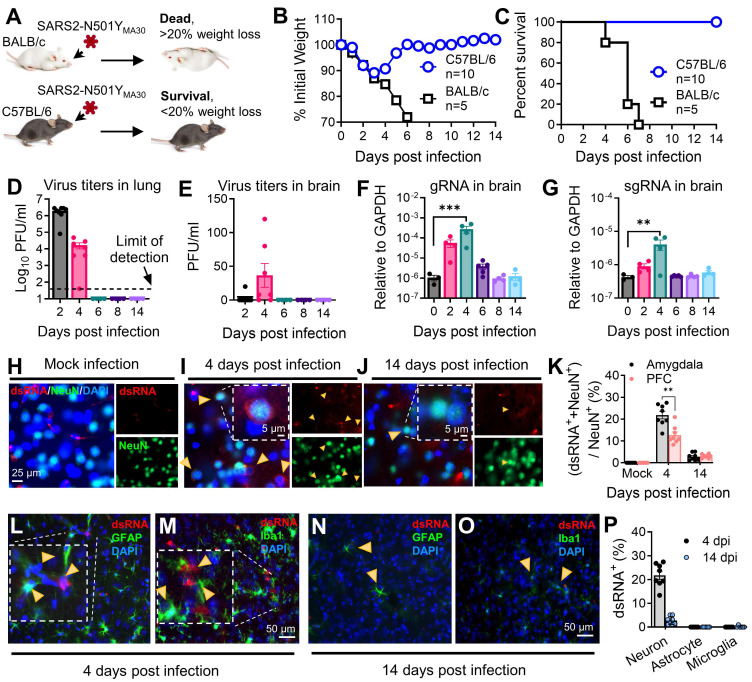
** Outcomes of intranasal infection with SARS2-N501Y_MA30_. (A)** Schematic depicting the outcomes of infection in young BALB/c and C57BL/6 mice following administration of 10^4^ PFU of SARS2-N501Y_MA30_. **(B-C)** Daily monitoring of body weight (B) and survival (C) in young BALB/c and C57BL/6 mice post-infection. **(D-E)** Virus titers in the lungs (D) and brains (E) of C57BL/6 mice infected with SARS2-N501Y_MA30_ at the indicated dpi. **(F-G)** Viral genomic RNA (gRNA) (F) and subgenomic RNA (sgRNA) (G) levels in brain tissues from SARS2-N501Y_MA30_ infected C57BL/6 mice. The levels of viral gRNA and sgRNA were normalized to GAPDH and presented as 2^-ΔCT (n=4 or 5 mice per group). CT values for viral gRNA or sgRNA from mock-infected tissues were consistently greater than 35. Statistical significance: ***p=0.001 and **p=0.0017 were determined by ordinary one-way ANOVA. **(H-J)** Immunofluorescence staining targeting dsRNA (red), neuronal nuclear protein (NeuN, green), and nuclei (DAPI, blue) in amygdala brain slices collected from mice infected with SARS2-N501Y_MA30_ infection at 4 or 14 dpi (I-J), compared to mock infection (H). Arrows indicate dsRNA and NeuN-positive neurons. Inserts show enlarged single-cell images. **(K)** Percentage of dsRNA-positive cells (dsRNA^+^) in slices from the amygdala and the PFC. The peak of dsRNA^+^ cells is observed at 4 dpi. Statistical significance: **p=0.0012 was determined by a two-tailed unpaired Student's t-test. **(L-O)** Immunofluorescence targeting dsRNA (red), astrocyte (glial fibrillary acidic protein, GFAP, green) or microglia (Ionized calcium-binding adaptor molecule 1, Iba1, green), and nuclei (DAPI, blue) in amygdala slices from mice 4 or 14 dpi. (P) Comparison of dsRNA^+^ cells with NeuN^+^, GFAP^+^, and Iba1^+^ at 4 dpi. Data are presented as mean ± SEM.

The inherent low affinity of the ancestral viral spike (S) glycoprotein for mouse ACE2 (mACE2) renders mice naturally resistant to SARS-CoV-2 infection 21, 22. However, specific mutations in the viral spike protein, such as Q498Y, P499T, and N501Y, can enhance binding to mACE2, resulting in asymptomatic infection in mice 36. Balb/c mice were utilized for the initial isolation and adaptation of the SARS-CoV-2-N501Y_MA30_ strain due to their known susceptibility to coronavirus infections, facilitating successful serial passage 37. Through serial passages of a recombinant SARS-CoV-2/spike N501Y virus in Balb/c mice, we successfully isolated a highly virulent mouse-adapted SARS-CoV-2 strain, designated as SARS2-N501Y_MA30_ 26. Young C57BL/6 mice were selected for subsequent behavioral experiments due to their relatively lower susceptibility to the SARS-CoV-2-N501Y_MA30_ and developed significant lung disease at the chosen dose (10^4^ PFU) **(Figure 1A)**. At this dose, Balb/c mice exhibited lethal outcomes, precluding long-term post-infection behavioral studies. In addition, C57BL/6 mice are widely used in behavioral neuroscience research, providing a robust background for assessing anxiety- and depression-like behaviors.

We intranasally infected mice with 10^4^ PFU/mouse of SARS2-N501Y_MA30_ 26_._ Comparable to our previous findings, young Balb/c mice developed lethal disease, whereas young C57BL/6 mice only displayed minimal weight loss of less than 20% and swift recovery within approximately one week **(Figure 1B-C)**. In C57BL/6 mice, viral titers peaked at 2 dpi in the lungs, followed by the presence of viral titers in the brain peaking at 4 dpi **(Figure 1D-E)**. Consistent with the observation, both viral genomic RNA (gRNA) and subgenomic RNA (sgRNA) were identified in the lung **(Figure S1)** and brain **(Figure 1F-G)**. Immunofluorescence targeting double-stranded RNA (dsRNA) further validated virus RNA replication presence in amygdala brain slices at 4 dpi, which subsequently disappeared by 14 dpi **(Figure 1H-J)**, with similar observations in the PFC, albeit with slight distinctions **(Figure 1K)**. These results collectively suggest direct viral infiltration into the brain, alongside respiratory infection. Remarkably, the production of infectious SARS-CoV-2 appeared to be controlled in the brain, as the titer is extremely low **(Figure 1E)**, and it did not induce major pathological changes within brain tissues **(Figure S2)**. Further investigation revealed that SARS-CoV-2 replication was predominant in neuron cells in the amygdala, rather than in microglia or astrocytes at both 4 dpi **(Figure 1L-M)** and 14 dpi **(Figure 1N-O)**. The summary of the percentages of dsRNA^+^ individual neurons, astrocytes, and microglia were compared at 4 dpi and 14 dpi **(Figure 1P)**. These findings indicated a SARS2-N501Y_MA30_ neuroinvasion with a peak at 4 dpi followed by a quick clearance.

Histological analysis of lung samples revealed acute lung injury using American Thoracic Society (ATS) guidelines for experimental acute lung injury (ALI) based on neutrophil infiltration in alveolar and interstitial space, hyaline membranes, alveolar wall thickening, and proteinaceous debris deposition 32. SARS-CoV-2 infection induced mild lung injury which peaked on day 6, accompanied by viral antigen presence at 2 dpi and disappeared after 6 dpi **(Figure S3)**. However, these changes, including infiltration and viral presence, resolved after a 14-week period. To explore host defense responses in SARS-CoV-2-infected mice, we assessed cytokine/chemokine gene expression in the lungs **(Figure S1)**. Notably, these genes exhibited significant upregulation at 2 dpi or 4 dpi, gradually subsiding to baseline levels after 6 dpi. These findings collectively illustrate a mild respiratory disease in the infected mice, which subsequently resolved within 14 days post-infection.

### SARS2-N501Y_MA30_ infection induces significant changes in anxiety- and depression-like behaviors

**Figure 2:**
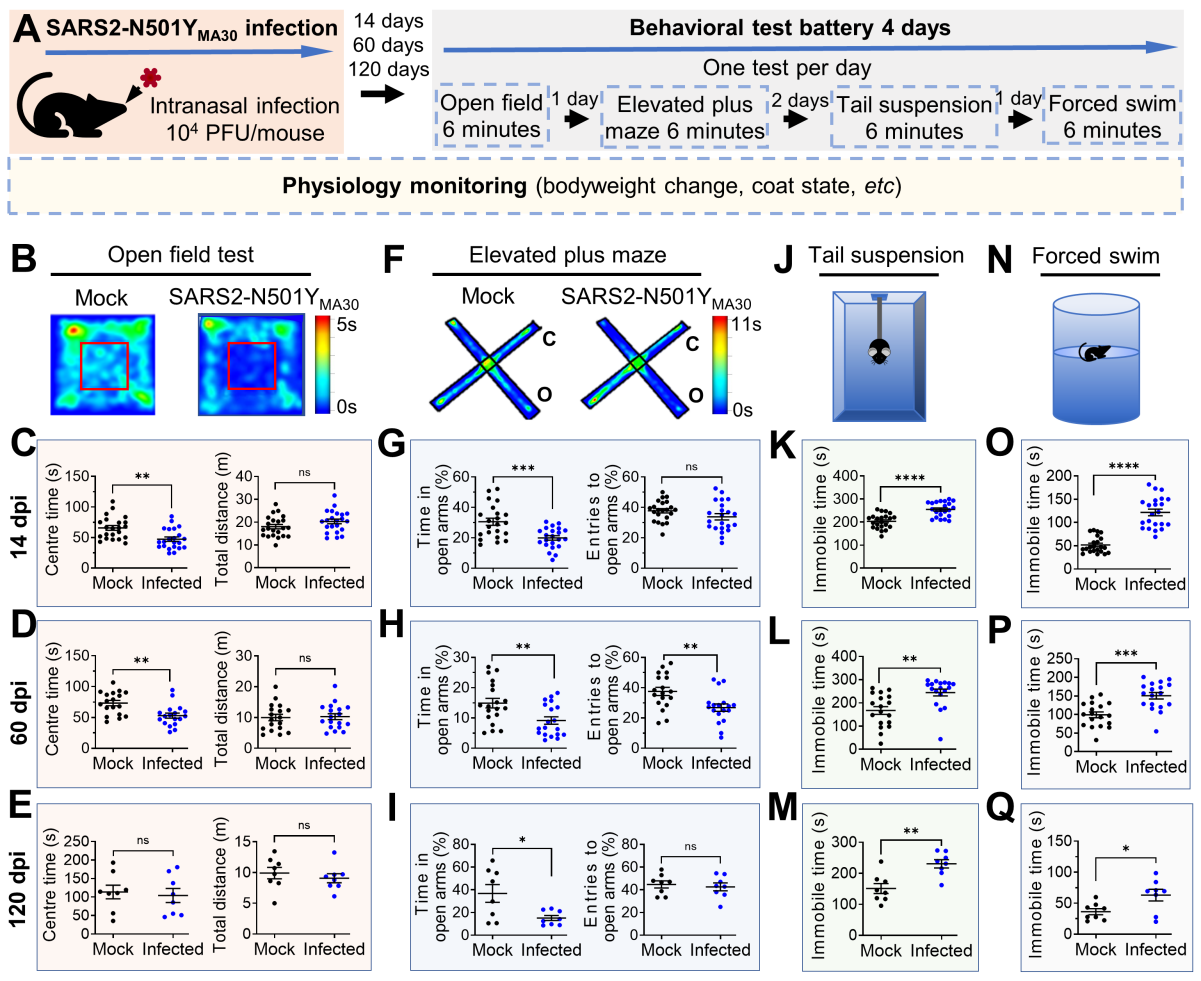
** Induction of anxiety- and depression-like behaviors in mice following SARS2-N501Y_MA30_ infection. (A)** Experimental design and timeline illustrating the administration of SARS2-N501Y_MA30_ and the behavioral test battery. **(B-E)** Open field test. Top: Representative heat map tracking of activity in the mock and SARS2-N501Y_MA30_ infected mice. Bottom: The total travel distance and time spent in the center area are shown for 14 dpi (C), 60 dpi (D), and 120 dpi (E), respectively. Statistical significance: 14 dpi group: **p=0.0013; ns, non-significant, p=0.1119; n=22 mice each group. 60 dpi group: **p=0.0017; ns, p=0.8427; n=18 mice in each group. 120 dpi group: ns, p=0.7272; p=0.4923; n=8 mice in each group. **(F-I)** Elevated plus maze test. Top: Representative heat map tracking of activity in mock and SARS2-N501Y_MA30_ infected mice in closed arms (c) and open arms (o). Bottom: The percentages of time spent in the open arms and the number of open arm entries are shown for 14 dpi (G), 60 dpi (H), and 120 dpi (I), respectively. Statistical significance: 14 dpi group: ***p=0.0004; ns, p=0.1241; n=22 mice each group. 60 dpi group: **p=0.0064; p=0.0059; n=18 mice in each group. 120 dpi group: *p=0.0187; ns, p=0.2243; n=8 mice in each group. **(J-M)** Schematics of the tail suspension test and results showing immobile time at 14 dpi (K), 60 dpi (L), and 120 dpi (M), respectively. Statistical significance: 14 dpi group: ****p<0.0001, n=22 mice in each group. 60 dpi group: **p=0.0015, n=18 mice in each group; 120 dpi group: **p=0.0018, n=8 mice in each group. **(N-Q)** Schematics of the forced swim test and results showing immobile time at 14 dpi (O), 60 dpi (P), and 120 dpi (Q), respectively. Statistical significance: 14 dpi group: ****p<0.0001; n=22 mice in each group. 60 dpi group: ***p=0.0001; n=18 mice in each group. 120 dpi group: *p=0.0245; n=8 mice in each group. All statistical analyses were performed using a two-tailed unpaired Student's t-test. Data are presented as mean ± SEM.

Our investigation successfully detected viral dsRNA within the amygdala **(Figure 1K)**, an area pivotal in coordinating defensive responses in mice 38 and emotional behaviors in humans 27. This finding prompted us to postulate that the infection may still exert influence over the functionality of this region and consequently modify the defensive behaviors of infected mice. To evaluate the impact of SARS-CoV-2 infection on anxiety- and depression-like behaviors, we conducted a well-established behavioral test battery in mice fourteen days after intranasal administration of SARS2-N501Y_MA30_ (10^4^ PFU/ mouse) **(Figure 2A)**. We initially assessed anxiety and depression-like behaviors at 14 dpi, a time point following viral clearance and resolution of acute symptoms (at 6 dpi)** (Figure 1B)**, to examine the onset of post-acute sequelae. No significant abnormalities in general health were observed in infected mice at 14 dpi, as compared to mock-infected controls.

In the OFT **(Figure 2B)**, the infected mice exhibited a decreased duration spent in the center area compared to the mock infection group, indicative of heightened anxiety-like behaviors for both male and female mice **(Figure 2C and Figure S4B, left)**. Both the SARS2-N501Y_MA30_ and mock infection groups demonstrated similar locomotor activity, implying a complete recovery of locomotion post-infection **(Figure 2C and Figure S4B, right)**. To assess the duration of persistent behavioral changes and potential long-term sequelae, we conducted further evaluations at 60 and 120 dpi in both male and female mice. At 60 dpi, behavioral deficits remained evident, with sustained anxiety-like phenotypes observed in the OFT **(Figure 2D)**. However, anxiety-like behaviors were not observed in the 120 dpi group **(Figure 2E)**.

In the EPM test **(Figure 2F)**, mice in the 14 dpi group significantly reduced their time spent in the open arms **(Figure 2G, left)**, while the number of entries into the open arms remained unaltered **(Figure 2G, right)**, further substantiating an augmentation in anxiety-like behaviors. Similar phenotypes were observed in both the 60 dpi **(Figure 2H)** and 120 dpi **(Figure 2I)** groups, supporting the persistence of anxiety-like behaviors as long-term sequelae. Consistent results were observed in the tail suspension **(Figure 2J)** and forced swim **(Figure 2N)** tests, both widely used to assess depression-like behaviors. Mice in all virus-infected groups (14 dpi, 60 dpi, and 120 dpi) exhibited increased immobility time compared to the mock groups **(Figure 2K-Q)**. To examine sex-dependent behavioral changes in detail, we analyzed mice's behaviors in the EPM, TST, and FST at 14 dpi **(Figure S4B-E)**. Our data suggest that both males and females exhibit similar anxiety-like and depression-like behaviors across all tests. While these behavioral tests do not account for all possibilities, the results may indicate a non-sex-specific behavioral adaptation to chronic viral effects.

These findings substantiate our conclusion that SARS2-N501Y_MA30_ infection leads to an escalation in anxiety- and depression-like behaviors in mice, indicating potential repercussions on brain function that warrant further exploration. Although these phenotypes slightly weaken by four months, our SARS2-N501Y_MA30_ -induced PASC model provides a sufficient timeframe to investigate underlying neuronal mechanisms.

### Temporal gene signatures in the brain after SARS-CoV-2 infection

**Figure 3:**
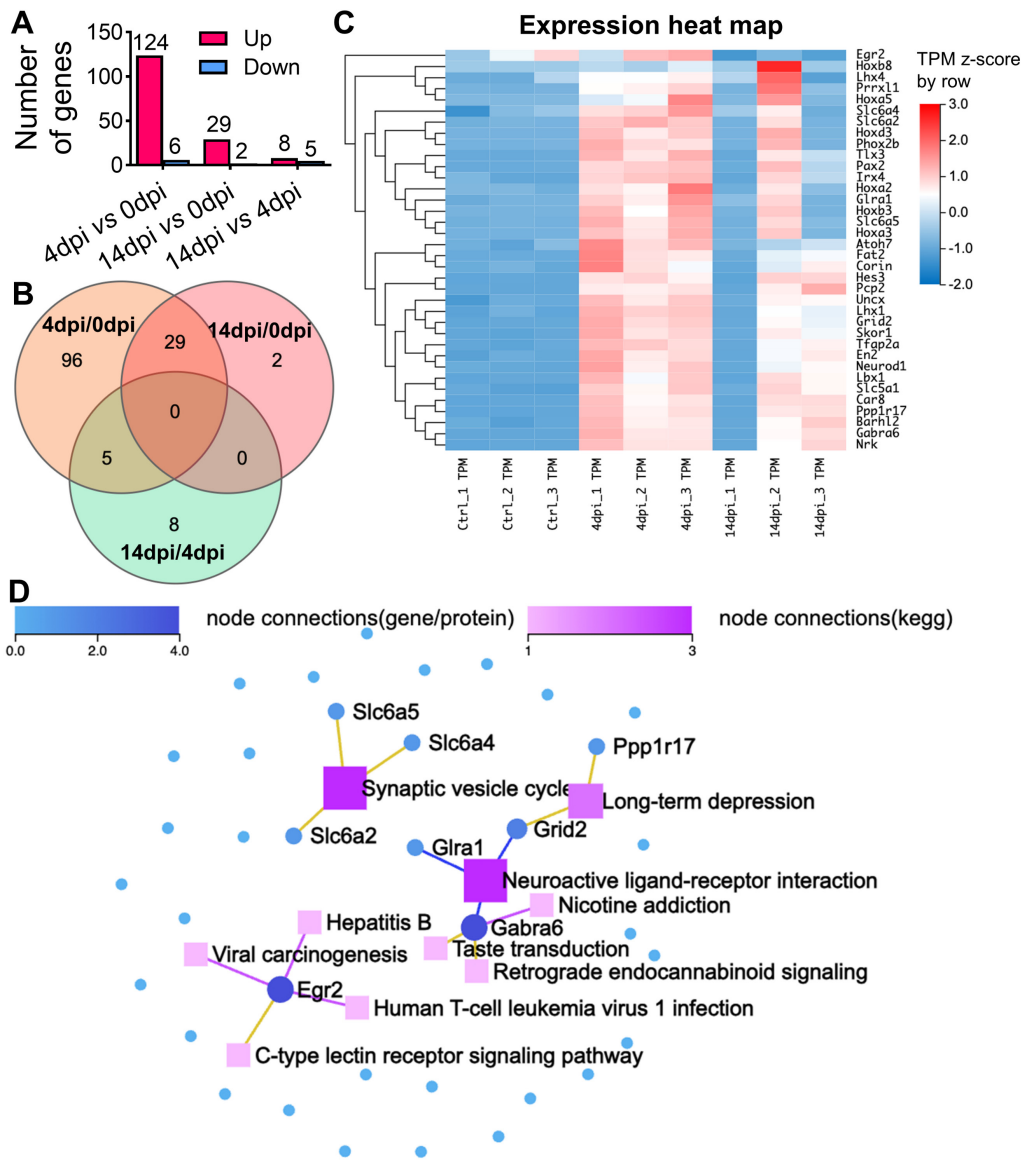
** RNA sequencing reveals distinct alterations in the brains of SARS2-N501Y_MA30_ infected mice. (A)** The histogram represents the corresponding numbers of differentially expressed genes (DEGs) between groups. (|log2fold| ≥ 1.5, Q value ≤ 0.05). **(B)** Venn diagram illustrating the differential gene expression analysis between groups of gene sets. The intersections show shared gene expression patterns, while non-overlapping regions indicate uniquely expressed genes. The numbers within each section denote the count of genes. **(C)** Heatmap displaying hierarchical clustering of 36 selected genes associated with neuron functions. **(D)** KEGG Network analysis of key driver genes and enriched KEGG pathways among the 36 selected genes. Dots symbolize genes, squares represent KEGG pathways, and lines connecting genes to squares signify gene enrichment within the respective pathway.

To further elucidate the molecular mechanisms associated with the observed neuroinvasion and subsequent behavioral alterations, we investigated the temporal dynamics of host transcriptional responses in the brain. This was achieved through bulk RNA sequencing (RNA-seq) on total brain RNA from both mock-infected animal cohorts (0 dpi) and animals infected with SARS2-N501Y_MA30_ at 4 dpi, and 14 dpi, respectively **(Figure 3 and Figure S5)**. Principal component analysis (PCA) unveiled distinct differences among mock and infected groups with samples from both 4 and 14 dpi **(Figure S5A).** The volcano map of differentially expressed genes (DEGs) revealed significant changes in host genes compared to mock-infected animals or between 4 and 14 dpi based on |log2fold| ≥ 1.5, Q value ≤ 0.05 **(Figure S5B)**. In the 4 dpi vs 0 dpi group, 124 genes were upregulated and 6 were downregulated, while in the 14 dpi vs 0 dpi group, 29 genes were upregulated and 2 were downregulated **(Figure 3A)**. There were only 8 genes upregulated and 5 downregulated between 4 and 14 dpi. Notably, 29 upregulated genes in the 14 dpi group also appeared in the 4 dpi group, many of which were associated with synaptic transmission and neuronal development **(Figure 3B and Figure S5).** Overall, there were 140 genes significantly changed **(Figure S5)**.

We performed pathway analysis based on the KEGG and Gene Ontology (GO) based enrichment analysis on the 140 genes **(Figure S5C and S5E).** Among them, 36 genes were implicated to be involved in different neuronal functions in the brain **(Figure 3C),** such as synaptic transmission, including Car8, Gabra6, Pcp2, Slc5a1, Tfap2a, or neuronal development, such as Barhl2, Hoxa5, Ppp1r17, Skor1, Fat2, and Nrk. We conducted a KEGG network analysis to delineate the interconnections among the 36 identified genes and identified genes related to synaptic vesicle cycle (slc6a2, slc6a4, and slc6a5), long-term depression (Ppp1r17 and Grid2), and neuroactive ligand-receptor interaction (Grid2, Glra1, and Gabra6) **(Figure 3D).** In conclusion, our comprehensive RNA-seq analysis has unveiled significant changes in host gene expression following viral infection. These alterations suggest a reprogramming of neuronal transcriptional states, corroborating the observed behavioral modifications. Moreover, the data indicates a profound influence on synaptic function and neurodevelopmental processes, underscoring the far-reaching effects of SARS-CoV-2 on the central nervous system.

### SARS2-N501Y_MA30_ infection induces spine remodeling in the amygdala

**Figure 4:**
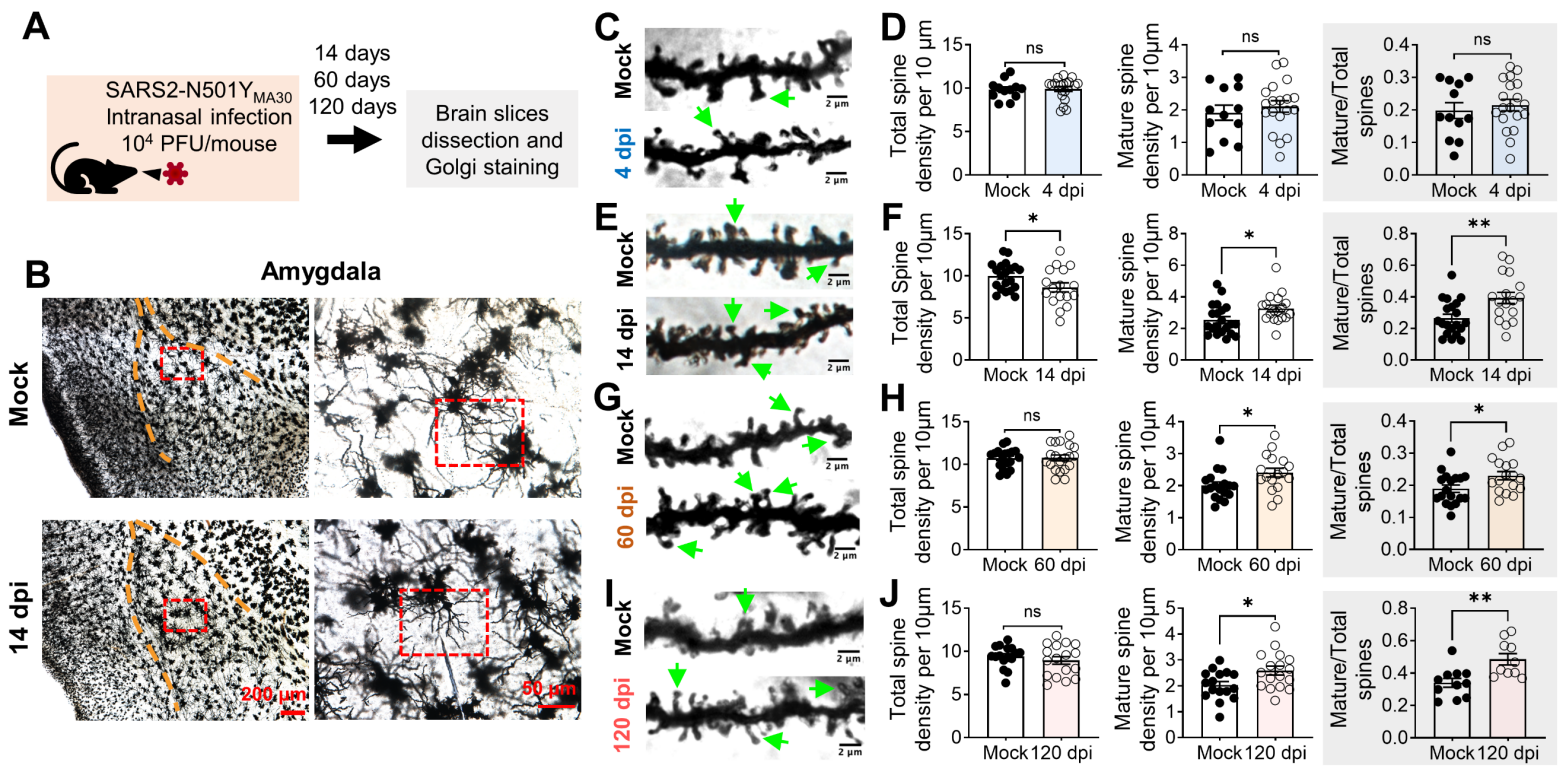
** Golgi-Cox staining of neurons in the amygdala of SARS2-N501Y_MA30_ infected mice. (A)** Experimental design and timeline depicting the administration of SARS2-N501Y_MA30_, dissection of brain slices, and Golgi staining process. **(B)** Left: Representative Golgi-Cox staining images of amygdala slices from mock-infected (top) and mice infected with SARS2-N501Y_MA30_ at 14 dpi (bottom). Right: Enlarged images from the red boxes in the left images for each respective group. **(C-D)** Representative images of dendrites in amygdala slices from mock-infected mice (top) and SARS2-N501Y_MA30_-infected mice at 4 dpi (bottom) (C). Green arrows indicate the mushroom spines. Comparison of total spine density, mature spine density, and the percentage of mature spines (the number of mature spines divided by the total number of spines) between the mock-infected and SARS2-N501Y_MA30_-infected groups (D). Statistical significance: ns, p=0.8482; p=0.5094; p=0.5734. n=12-19 neurons/4 mice in each group. **(E-F)** Representative images of dendrites at 14 dpi (bottom) (E) and spine comparisons (F). Statistical significance: *p=0.0427; *p=0.0216; **p=0.0049. n=17-21 neurons/4 mice in each group. **(G-H)** Representative images of dendrites at 60 dpi (bottom) (G) and spine comparisons (H). Statistical significance: ns, p=0.8948; *p=0.0308; **p=0.0250. n=17-20 neurons/4 mice in each group. **(I-J)** Representative images of dendrites at 120 dpi (bottom) (I) and spine comparisons (J). Statistical significance: ns, p=0.4205; *p=0.0177; **p=0.0038. n=10-17 neurons/4 mice in each group. Statistical analysis was performed using a two-tailed unpaired Student's t-test. Data are presented as mean ± SEM.

To complement the gene expression changes revealed by our RNA-seq analysis, we next examined the morphological alterations in neurons within the amygdala, a region implicated in depression-related behaviors. This investigation aimed to confirm the impact of SARS2-N501Y_MA30_ infection on synaptic plasticity through the observation of dendritic spine remodeling. Dendritic morphology has been widely implicated in the mechanisms governing synaptic plasticity 35, 39, 40. Dendritic spines constitute the principal target of neurotransmission input within the CNS 41, and their density and structure form the foundation for physiological alterations in synaptic efficacy that underlie learning and memory processes 42. We hypothesized that dendritic structure and plasticity undergo alterations following SARS2-N501Y_MA30_ infection. At 14, 60, or 120 days after SARS2-N501Y_MA30_ infection, brain slices containing the amygdala were meticulously dissected, fixed, and subjected to Golgi staining to label the spines **(Figure 4A)**. Neurons were randomly selected from within the basolateral amygdala, with a focus on secondary branches located 50-100 µm away from the soma **(Figure 4B)**. Subsequently, spine morphology was manually visualized and analyzed **(Figure 4C-I)**.

To characterize spine morphology, we categorized dendritic processes into two distinct morphological classes: mature and immature spines 43. Mature spines, predominantly exhibiting a "mushroom-like" morphology, exhibit more stable postsynaptic structures and are considered functional spines. Conversely, immature spines, characterized by their thin, stubby, and filopodial features, represent unstable postsynaptic structures with transitional properties. Immature dendritic spines are believed to hold the potential for future synaptic plasticity, either maturing into functional spines or disappearing from the dendrite 44. Spine categories were identified based on parameters described in previous studies and methods 35, 45.

To elucidate the time-dependent synaptic changes following viral infection, we analyzed the dynamic alterations in dendritic spine structure within the amygdala of SARS2-N501Y_MA30_-infected mice at 4, 14, 60, and 120 dpi. Quantitative morphometric analysis revealed temporally distinct patterns of spine remodeling. Neither total dendritic spine density (control: 9.822 ± 0.3167 spines/10 μm; infected: 9.908 ± 0.2891spines/10 μm) nor mature mushroom-type spine density (control: 1.915 ± 0.2316 spines/10 μm; infected: 2.108 ± 0.176 spines/10 μm) showed significant alterations at 4 dpi **(Figure 4C-D)**, suggesting that 4 dpi, the peak of brain neurotropism, is too early to detect synaptic plasticity alterations. However, the percentage of mature spines relative to the total spines remained elevated at 14 dpi **(Figure 4E-F)**, 60 dpi **(Figure 4G-H)**, and 120 dpi **(Figure 4I-J)**.

These findings are not surprising, as 4 dpi marks the time when neurons appear to begin being affected by viral infection via nasal application **(Figure 1)**. Our data suggests that direct targeting of neurons by SARS2-N501Y_MA30_ may not immediately alter (damage or promote) synapses. Instead, viral targeting of neurons may trigger the release of factors that activate glial cells, such as microglia, which in turn modulate neuronal synapses. This mechanism may explain the observed dendritic differences at 14 dpi.

### Spike protein increases microglia-dependent fEPSPs in amygdala slices

**Figure 5:**
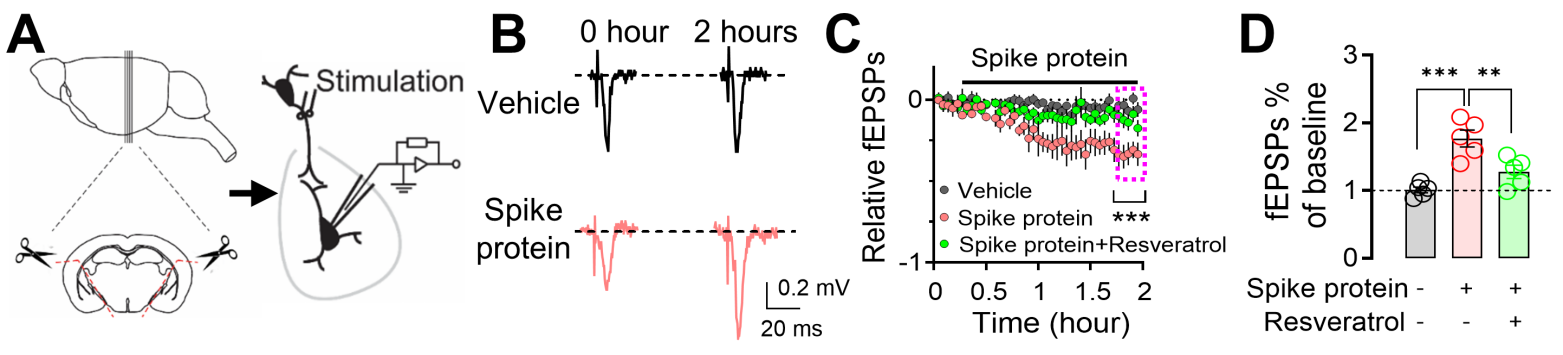
** Enhancement of microglia-dependent fEPSPs in the amygdala by SARS-CoV-2 variant B.1.351 spike protein. (A)** Schematic representation of fEPSP recordings in the amygdala brain slices. **(B)** Representative fEPSP traces were recorded at the beginning (0 hours) and the end of the 2-hour recordings, during perfusion with either vehicle or spike protein (S1+S2, B.1.351, β variant, 167 ng/ml). **(C)** Average fEPSP data. The spike protein was applied to the slice (167 ng/ml) at the indicated time point (red trace). In the vehicle group, a mock aqueous buffer solution without the spike protein was used (gray trace). The enhancement of fEPSPs by the spike protein was attenuated by pre-treating brain slices with 50 μM Resveratrol (green trace). **(D)** Summarized amplitudes of the last five fEPSPs as shown in (C). Statistical significance: ***p=0.0003 and **p=0.0082 were determined by one-way ANOVA with Tukey's post hoc multiple comparisons. The analysis includes data from 5 slices in each group. Data are presented as mean ± SEM.

Exposure to the SARS-CoV-2 spike protein has emerged as a topic of interest in neurobiology, particularly regarding its impact on neuronal function. Previous research has provided initial insights into how spike protein can influence neuronal morphology and function 31, 46, 47. To further explore the mechanistic aspects of this phenomenon, we focused on the amygdala, a region critically involved in emotional processing and defensive behaviors. We perfused amygdala slices from mice with the recombinant B1.351 variant spike protein (167 ng/ml) 31, which has a high affinity to mouse ACE, and monitored changes in fEPSPs for 2 hours **(Figure 5A)**. We observed a significant increase in fEPSPs following exposure to the B1.351 variant spike protein, indicating heightened synaptic transmission and neuronal excitability **(Figure 5B-C)**. Despite the fact that whether SARS-CoV-2 spike protein increases or decreases neuronal activity is controversial 31, 46-48, this observation is noteworthy, as it suggests that the spike protein, when directly interacting with neurons, has the potential to amplify their responsiveness to incoming signals. This heightened neuronal response could, in turn, render individuals more sensitive to various stimuli, potentially leading to alterations in behaviors or physiological reactions. Interestingly, the administration of 50 μM Resveratrol, a known inhibitor of microglial activation 49, effectively counteracted the spike protein-induced enhancement of fEPSPs **(Figure 5C-D)**. This finding suggests that microglia, the intrinsic resident immune sentinels of the brain, may play a pivotal role in mediating the effects of the spike protein on neuronal activity.

### Microglia activation after SARS-CoV-2 infection or exposure to spike protein

**Figure 6:**
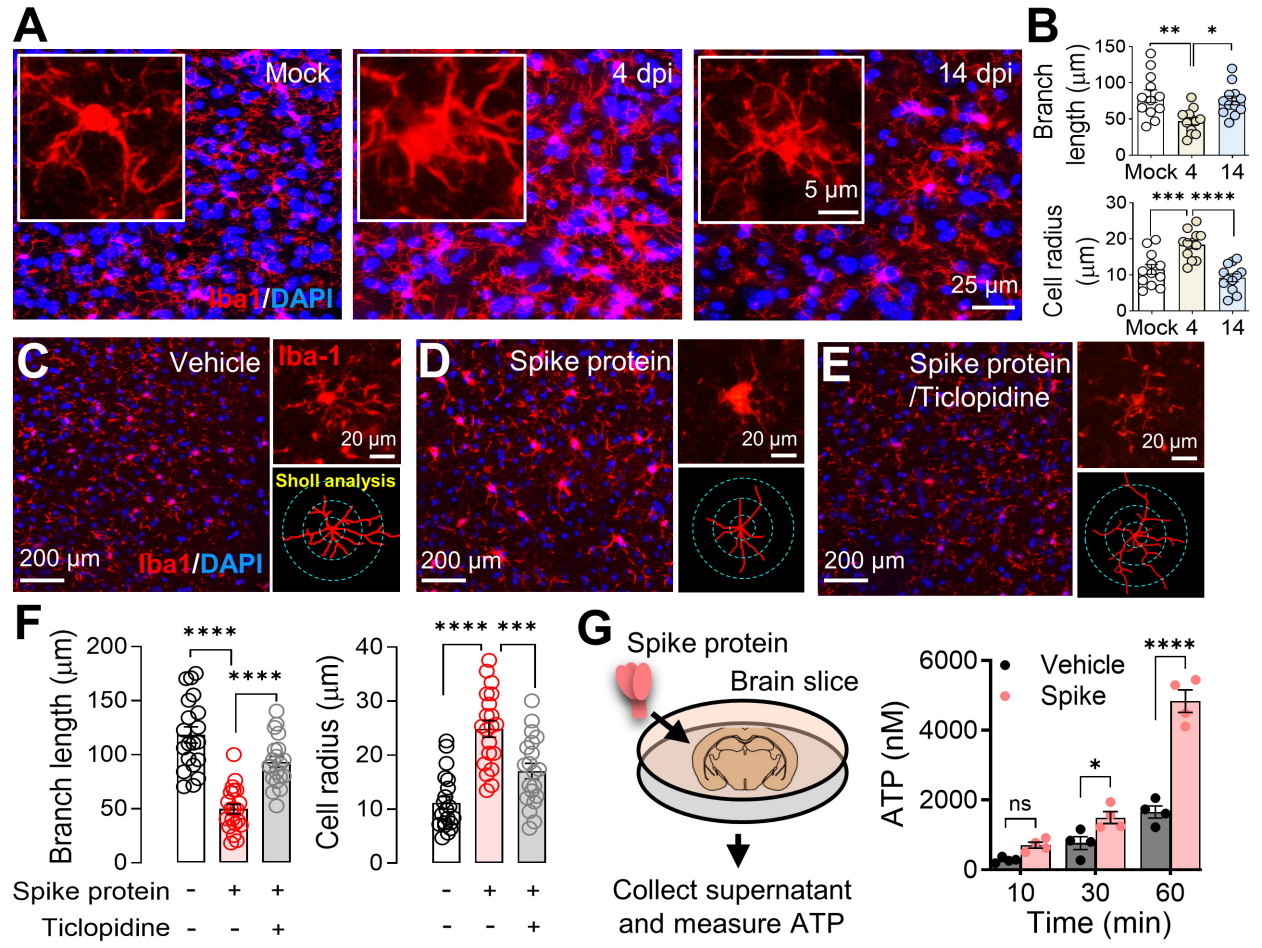
** Microglial activation in response to SARS-CoV-2 infection and B1.351 spike protein. (A)** Dynamics of microglial activation post-infection in the amygdala of mice. Immunofluorescence staining for Iba1 highlights microglia in amygdala slices from mice at indicated time points following infection. An enlarged view of individual microglia is provided to detail the changes in shape and morphology indicative of activation states. **(B)** Microglial activation results from (A) were compared using Sholl analysis, which quantifies total branch length and cell area radius. Statistical significance: **p=0.0027, *p=0.0143, ***p=0.0007, and ****p<0.0001 were by one-way ANOVA with Tukey's post hoc multiple comparisons. The analysis includes data from 12 cells across 4 slices in each group. **(C-E)** Microglial response to perfusion with the spike protein. Immunostaining with Iba-1 illustrates microglial morphology in brain slices after a one-hour perfusion with the (B) vehicle or (C) spike protein (S1+S2, B.1.351, β variant, 167 ng/ml), and (D) spike protein following a 30-minute pretreatment with 50 μM Ticlopidine, a P2Y12R antagonist. The series showcases the microglial response to the spike protein, with additional magnified images highlighting the individual microglia and their morphological analysis using Sholl analysis, presented to the right of each panel. **(F)** Microglial activation results from (C-E) were compared using Sholl analysis, which quantifies total branch length and cell area radius. Statistical significance: ****p<0.0001 and ***p=0.0006 were by one-way ANOVA with Tukey's post hoc multiple comparisons. The analysis includes data from 30 cells across 4 slices in each group. **(G)** Quantification of ATP release. Brain slices were incubated in cold PBS with either vehicle or spike protein (167 ng/ml). Supernatants were collected at 10, 30, and 60 minutes post-incubation. The amount of ATP released into the supernatant was quantified. Each dot is an independent brain slice and represents the mean of 2 technical replicates. Statistical significance: *p=0.0374 and ****p<0.0001 were determined by two-way ANOVA. Data are presented as mean ± SEM.

Following the discovery in **Figure 5**, we conducted further investigations into microglial reactivity during SARS2-N501Y_MA30_ infection. Through immunofluorescence staining for Iba1, we documented a significant activation of microglia, evidenced by their morphological shift from ramified to amoeboid shapes 50. This shift was notable at 4 dpi, and by 14 dpi, microglia activity had reverted to the baseline quiescent state observed in the mock infection group, as illustrated in **Figure 6A-B**. This finding provides further support for the hypothesis that microglial activation may contribute to the observed alterations in neuronal activities within the amygdala following SARS-CoV-2 infection.

Microglia, the brain's resident immune cells, are highly dynamic, engaging with neurons and other brain cells upon activation. Their processes reach out to neighboring cells, forming a complex network of interactions crucial for understanding the neuroimmune impact of SARS-CoV-2 infection 51. To further elucidate the mechanism by which the spike protein promoted microglia activation, we visualized microglia by immunofluorescence staining for Iba1 in mouse amygdala slices. Our observations confirmed the activation of microglia following SARS-CoV-2 B1.351 spike protein perfusion **(Figure 6C-D)**. Interestingly, the activation was attenuated by the purinoceptor P2Y_12_ (P2Y12R) antagonist ticlopidine **(Figure 6D-F)**, indicating that the P2Y12R signaling pathway mediates this response. P2Y_12_, selectively expressed in microglia 52, serves as the main adenosine triphosphate (ATP) receptor, initialing microglial activation and mobilization in response to ATP, a potent neuronal chemoattractant released from neurons 53, 54. Additionally, it is worth noting that SARS-CoV-2 infection has been shown to induce ATP release from host cells, including neural cells, via specific channels 55, 56, a phenomenon we corroborated by detecting elevated ATP levels in brain slices perfused with spike protein **(Figure 6G)**.

Spike protein has been detected circulating in the plasma of individuals with PASC for up to 12 months following initial diagnosis 57. To verify whether spike protein can also be detected in the plasma of infected mice in our model, we collected plasma from infected mice at 0, 2, 4, 6, 8, and 14 dpi and measured the spike protein concentration by ELISA. The peak spike protein level in plasma was observed at 2 dpi (474.8 ± 191.5 pg/ml, mean ± SEM), and subsequently gradually decreased **(Figure S6)**. After 8 dpi, spike protein was undetectable in plasma by ELISA. ELISA measurements for spike protein in whole brain lysates at 2-14 dpi were below the detection limit of 7.81 pg/ml. While this suggests limited spike protein in whole brain homogenates, the early detection of gRNA and sgRNA indicates a transient period of potential spike protein/virus production in specific CNS regions or blood. Future studies employing more sensitive and region-specific assays are needed to precisely map the location and timing of active virus replication and spike protein expression in the brain. We hypothesize that this early, potentially localized, encounter of spike protein or the virus with CNS cells triggers ATP secretion from neurons, subsequently activating microglia and influencing neuronal function.

### Brain immune inflammation and anti-viral responses during viral infection

**Figure 7:**
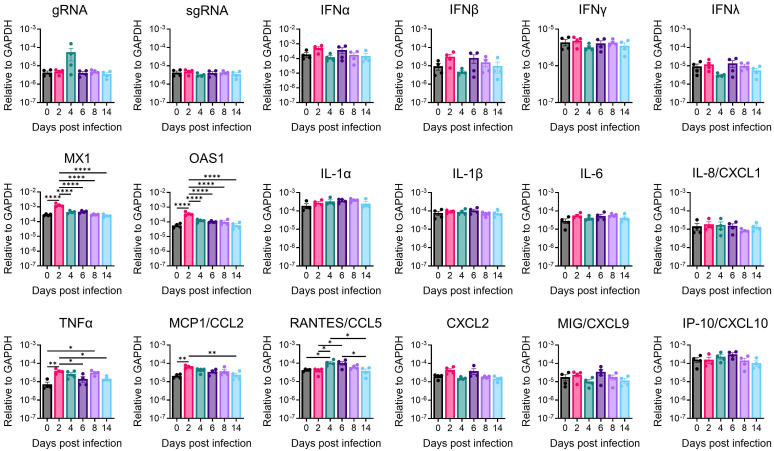
** Antiviral and inflammatory gene expression in the amygdala of SARS-CoV-2-infected mice.** Amygdala brain slices from C57BL/6 mice intranasally infected with 10^4^ PFU of SARS2-N501Y_MA30_ were collected at the indicated dpi. Viral gene, antiviral, and inflammatory transcripts were measured by qRT-PCR analyzing total RNA extracted from the amygdala of mock-infected (0 dpi) and infected young C57BL/6 mice. Each amygdala was collected from one individual mouse. Mock (0 dpi), 2, 4, 6, 8, 14 dpi: n=4. The levels of transcripts were normalized to GAPDH and presented as 2^-ΔCT. Statistical significance: *p<0.05, **p<0.01, and ****p<0.0001 were determined by ordinary one-way ANOVA. Data are presented as mean ± SEM.

Cytokines and chemokines serve as crucial indicators of anti-viral response and immune inflammation. To delineate the immune responses within the brain during SARS-CoV-2 infection, we conducted quantitative reverse-transcription PCR (RT-qPCR) to profile cytokines and chemokines expression across various time points in the amygdala and whole brain **(Figure 7 and Figure S7)**. In the amygdala, we successfully detected viral genomic RNA (gRNA); however, we were unable to detect subgenomic RNA (sgRNA), suggesting the absence of active viral RNA transcription in this specific brain region at detectable levels by our assay. Despite the lack of detectable sgRNA, we observed significant upregulation of antiviral genes, such as Mx1 and OAS1, along with chemokines monocyte chemoattractant protein-1 (MCP1/CCL2) and CCL5, and the pro-inflammatory cytokine TNFα, with peak expression generally at 2 or 4 dpi **(Figure 7)**. Notably, neither the amygdala nor the whole brain exhibited significant upregulation of interferon (IFN) genes (IFN-β, IFN-γ, IFN-λ) following SARS-CoV-2 infection in our model, in contrast to typical antiviral responses. However, indicating a broader inflammatory response in the whole brain, transcripts encoding pro-inflammatory cytokines, such as interleukin-1α (IL-1α), interleukin-6 (IL-6), interleukin-8 (IL8/CXCL1), and especially MCP1/CCL2, exhibited significant upregulation at 2- or 4-days post-infection **(Figure S7)**. These temporal increases coincided with the presence of viral RNA in the brain. Importantly, it is worth highlighting that the alterations observed in immune gene expression within the brain were modest in comparison to those observed in the lungs and returned to baseline levels after 6 days post-infection. This discovery underscores the presence of restrained immune inflammation and anti-viral responses within the brain. Our qPCR data implies distinct immune responses in different brain regions, indicating regional heterogeneity in the brain's reaction to SARS-CoV-2 infection. The potential role of these immune changes warrants further comprehensive investigations to unravel the underlying mechanisms responsible for observed behavioral alterations and identify potential targets for therapeutic intervention.

## Discussion

Currently, several major hypotheses explain the neurological and CNS symptoms in PASC 58-63. First, SARS-CoV-2 or its fragments (e.g., spike protein, RNA) may persist in the body, including the CNS, forming a "reservoir" that triggers ongoing immune activation and tissue damage. Second, acute infection induces inflammatory and immune responses that fail to fully resolve, leading to prolonged CNS impact. Third, SARS-CoV-2 may trigger autoimmune responses, causing the immune system to attack CNS tissues. Fourth, vascular damage, including microvasculature dysfunction, impairs blood flow and oxygen supply, affecting brain function. Fifth, viral infection may disrupt cellular metabolism and mitochondrial function, leading to energy deficits in neurons and glial cells. Studying these mechanisms directly in humans is challenging. To address this, we refined our mouse-adapted SARS-CoV-2 model to investigate PASC neurological sequelae, selecting a viral dose that induces significant but non-lethal disease in C57BL/6 mice, enabling long-term observation of neurological effects.

Our findings revealed the presence of SARS-CoV-2 RNA in the CNS following intranasal inoculation in a SARS2-N501Y_MA30_ infection mouse model **(Figure 1)**. These results are consistent with previous reports indicating that SARS-CoV-2 can invade the human brain 5, 6, supporting the notion of neurotropism. SARS-CoV-2 probably enters the CNS through two potential pathways 64. The first involves accessing the CNS via the neural-mucosal interface in the olfactory mucosa, enabling the virus to spread from the periphery olfactory neurons into the neurons of the olfactory bulb. The second pathway is through entry into the brain via blood circulation, potentially breaching the blood-brain barrier (BBB). In this scenario, the integrity of the BBB could be disrupted by inflammatory responses triggered by the infection.

Notably, following SARS-CoV-2 infection and CNS invasion, we observed significant anxiety- and depression-like behaviors in behavioral assays **(Figure 2)**. These findings support the hypothesis that viral persistence in the CNS may drive ongoing neuropathology and behavioral sequelae. However, we did not detect persistent viral RNA in the brain beyond the acute phase. Viral RNA peaked around 4 dpi in the whole brain and amygdala but fell below detection by 6 dpi. Despite this, transcriptomic changes emerged by 4 dpi, alongside dendritic spine alterations in the amygdala persisting at > 60 dpi. This suggests that even transient viral presences may trigger long-term effects. While our RT-qPCR assay did not detect viral RNA beyond 6 dpi, we cannot exclude the possibility that low levels of viral RNA or fragments persist below detection limits. Such persistent viral components might still induce a chronic, low-level inflammatory state that is below the detection threshold of our bulk tissue qRT-PCR method and contribute to ongoing pathology. Further studies employing more spatially resolved techniques, such as single-cell sequencing or targeted analysis of other specific brain regions, are needed to provide a more granular understanding of the neuroinflammatory landscape following SARS-CoV-2 infection.

Microglia and astrocytes, serving as the primary immune cells of the CNS, are critical regulators of brain homeostasis and frontline defenders against infections 65. Upon detecting pathogens or damage-associated signals, these cells transition rapidly from a surveillance state to an activated immune-responsive phenotype. In our study, robust microglial activation was observed in brain regions colocalized with viral RNA detection sites, implicating microglia in mediating SARS-CoV-2-induced neurobehavioral changes. Although microglia themselves do not appear to be direct targets of SARS-CoV-2 infection **(Figure 1M)** 7, 66, their activation is likely triggered by the initial viral invasion of neuronal cells. While our investigation centers on the SARS-CoV-2-neuron-microglia axis, we propose that future studies prioritize elucidating astrocyte-specific signaling pathways, which are equally vital for a complete understanding of SARS-CoV-2 neurotropism.

Our findings highlight the therapeutic potential of targeting neuroinflammatory pathways to alleviate anxiety- and depression-like symptoms in PASC. Anti-inflammatory and immunomodulatory drugs that reduce microglial activation are promising candidates for mitigating SARS-CoV-2-related neuropsychiatric effects. For example, Resveratrol, a neuroprotective polyphenol evaluated in vitro in this study, warrants further investigation for modulating microglial responses. Additionally, our transcriptomic data suggest other potential targets, including TNF-α or MCP-1/CCL2 inhibitors. However, further research is needed to identify key inflammatory mediators, clarify downstream signaling pathways, and rigorously assess therapeutic efficacy and safety in preclinical and clinical settings.

While our study provides valuable insights into SARS-CoV-2 neurotropism, microglial activation, neuronal activity, and anxiety- and depression-like behaviors, several limitations should be acknowledged. First, murine models cannot fully recapitulate human disease, as differences in lifespan, age, and strain-specific SARS-CoV-2 sensitivity may limit direct translation to human COVID-19 and PASC. Second, while our model exhibited mild respiratory disease, further research is needed to determine how varying severities of illness influence long-term behavioral changes and CNS immune responses, particularly in aged mice. Clinical data suggest a lower risk of long-term symptoms in asymptomatic cases 67, but the impact of respiratory severity remains an important area for future study. Third, while we observed CNS cytokine and chemokine upregulation, we did not assess the contribution of systemic inflammatory mediators to microglial activation and neuronal dysfunction. Notably, prior studies show that systemic inflammation, chemotherapy, and brain radiation can lead to persistent microglial activation 68-72. Additionally, a recent study in SARS-CoV-2 infected AAV-hACE2-sensitized mice and H1N1-infected mice found that even mild respiratory disease-activated white-matter-selective microglia, leading to oligodendrocyte loss, impaired neurogenesis, and elevated CCL11 levels in the brain 73.

Despite these limitations, the characterization of neurological sequelae following SARS-CoV-2 N501Y_MA30_ infection in mice provides a valuable pre-clinical model for dissecting the neuropathogenesis of PASC. Continued research using this and complementary models remains crucial for advancing our understanding of COVID-19's neurological impact and for developing effective therapeutic strategies to mitigate long-term neuropsychiatric consequences.

## Supplementary Material

Supplementary figures and table.
