# Supplementary material for: Investigating post-infection anxiety- and depression-like behaviors in a SARS-CoV-2 mouse model
Source: Theranostics. 2025 Apr 21;15(12):5738–55. doi: 10.7150/thno.102752 (PMC12068287; doi:10.7150/thno.102752)
Supplement: Supplementary file 1 — Supplementary figures and table. [file thnov15p5738s1.pdf]

Supplemental Figure S1

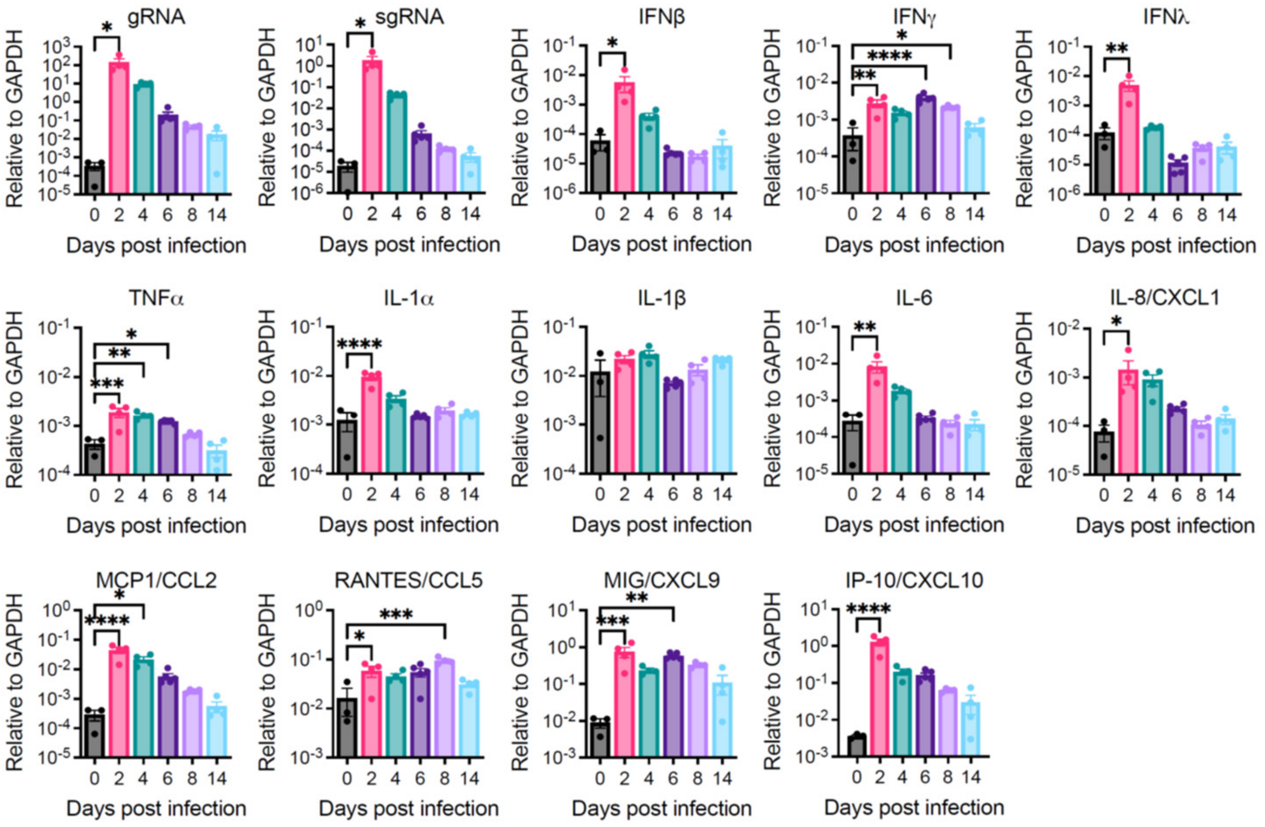

Figure S1. SARS2-N501Y$_{MA30}$ virus RNA replication and host immune responses in the lungs. Total RNA was extracted from lungs collected from C57BL/6 mice (n=3 to 5), intranasally infected with 10^4 PFU of SARS2-N501Y$_{MA30}$ at designated dpi. Viral RNA, as well as host cytokine and chemokine transcripts, were quantified using qRT-PCR. Viral gRNA or sgRNA CT values from mock-infected tissues (0 dpi) exceeded 35. Transcript levels were normalized to GAPDH and presented as 2^-ΔCT. Statistical significance was assessed using ordinary one-way ANOVA, with significance levels indicated as follows: *p<0.05, **p<0.01, ***p<0.001, and ****p<0.0001. Data are presented as mean ± SEM.

Supplemental Figure S2

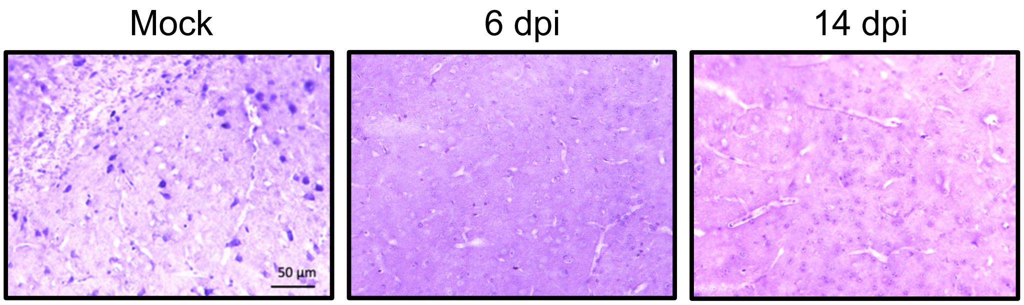

Figure S2. Brain histopathology after mock and SARS2-N501Y$_{MA30}$ infection. The representative images of brain tissue sections were stained with H&E staining at indicated days following either mock or SARS-CoV-2 infection. Scale bar: 50 µm.

Supplemental Figure S3

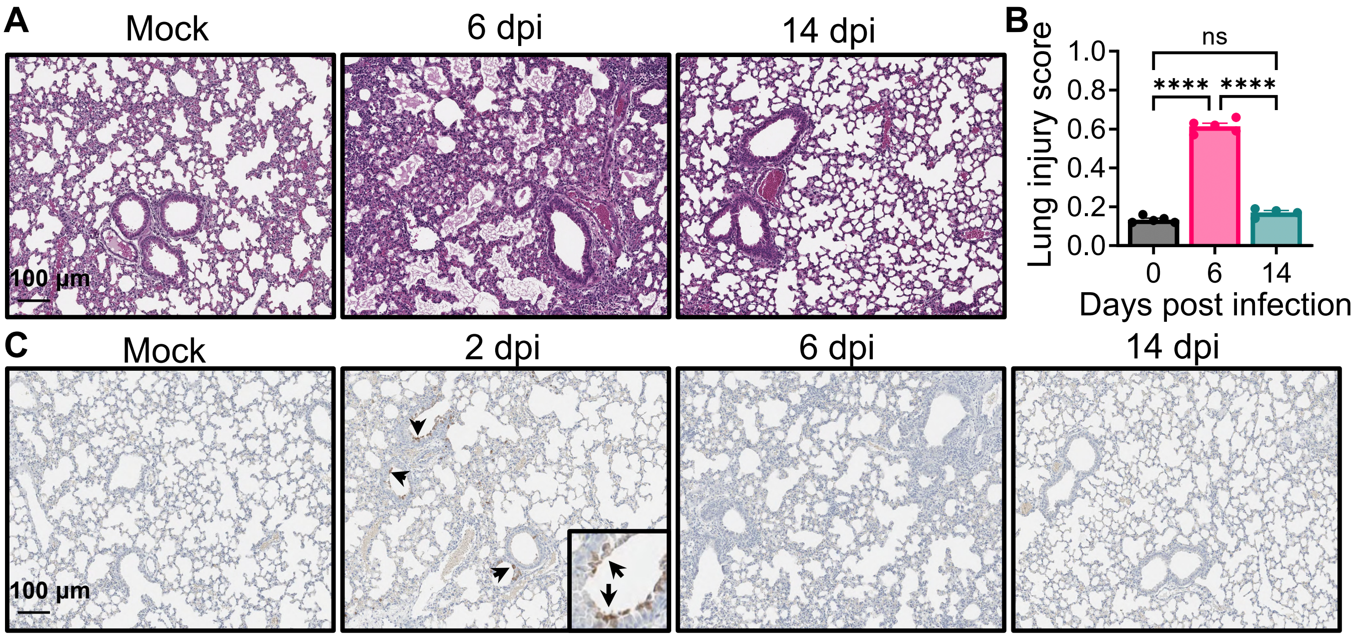

Figure S3. Lung pathology and immunohistochemistry of viral protein. (A) Lung sections from mice infected with SARS2-N501Y$_{MA30}$ (n=5 for mock infection and 6 dpi; n=4 for 14 dpi) underwent H&E staining. **(B)** Acute lung injury severity was assessed blindly following ATS guidelines [32], with 0 for no injury, 1 for mild to moderate injury, and 2 for severe injury. ****p<0.0001. Data are shown as mean ± SEM. **(C)** Lung tissue immunohistochemistry revealing SARS-CoV-2 N protein (black arrows) at specified dpi.

Supplemental Figure S4

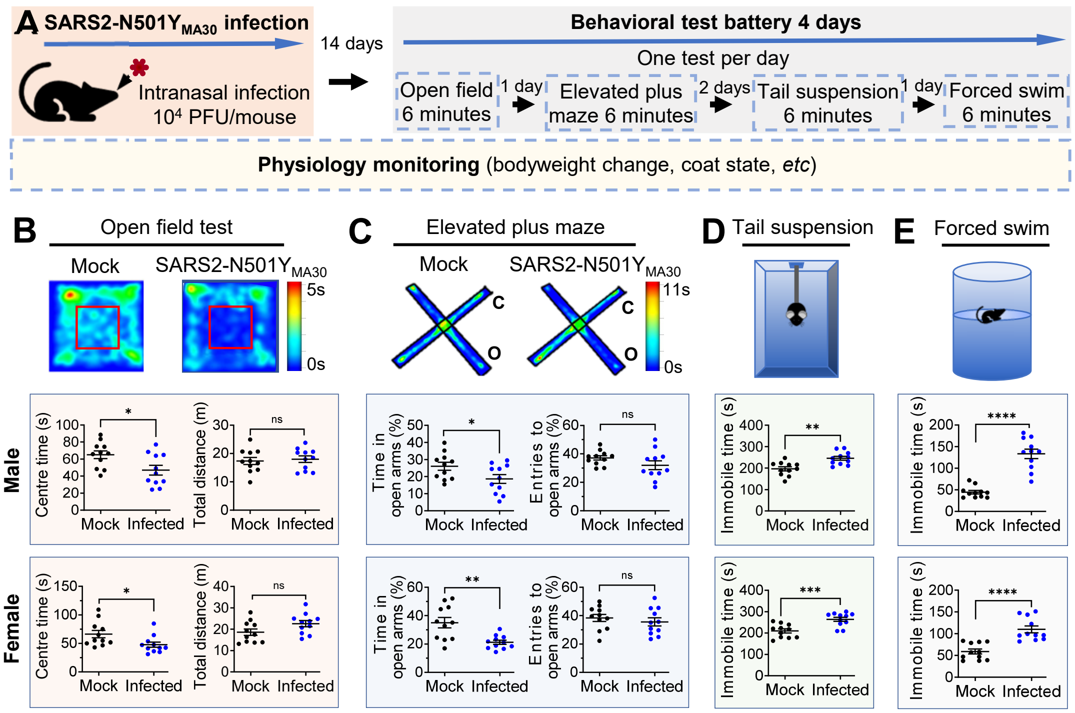

Figure S4. Gender dependent anxiety- and depression-like behaviors in mice following SARS2-N501Y$_{MA30}$ infection. (A) Experimental design and timeline illustrating the administration of SARS2-N501Y$_{MA30}$ and the behavioral test battery. **(B)** Open field test. Top: Representative heat map tracking of activity in the mock and SARS2-N501Y$_{MA30}$ infected mice. Bottom: The total travel distance and time spent in the center area are shown for males and females, respectively. Statistical significance: Male group: *p=0.0219; ns, p=0.7185; n=11 mice in each group. Female group: **p=0.0331; ns, p=0.0722; n=11 mice in each group. **(C)** Elevated plus maze test. Top: Representative heat map tracking of activity in mock and SARS2-N501Y$_{MA30}$ infected mice in closed arms (C) and open arms (O). Bottom: The percentages of time spent in the open arms and the number of open arm entries are shown for males and females, respectively. Statistical significance: Male group: *p=0.0457; ns, p=0.1487; n=11 mice in each group. Female group: **p=0.0022; ns, p=0.4732; n=11 mice in each group. **(D)** Schematics of the tail suspension test and results showing immobile time in males and females, respectively. Statistical significance: Male group: **p=0.0014; n=11 mice in each group. Female group: ***p=0.0007; n=11 mice in each group. **(E)** Schematics of the forced swim test and results showing immobile time in males and females, respectively. Statistical significance: Male group: ****p<0.0001; n=11 mice in each group. Female group: ****p<0.0001; n=11 mice in each group. All statistical analyses were performed using a two-tailed unpaired Student's t-test. Data are presented as mean ± SEM.

Supplemental Figure S5

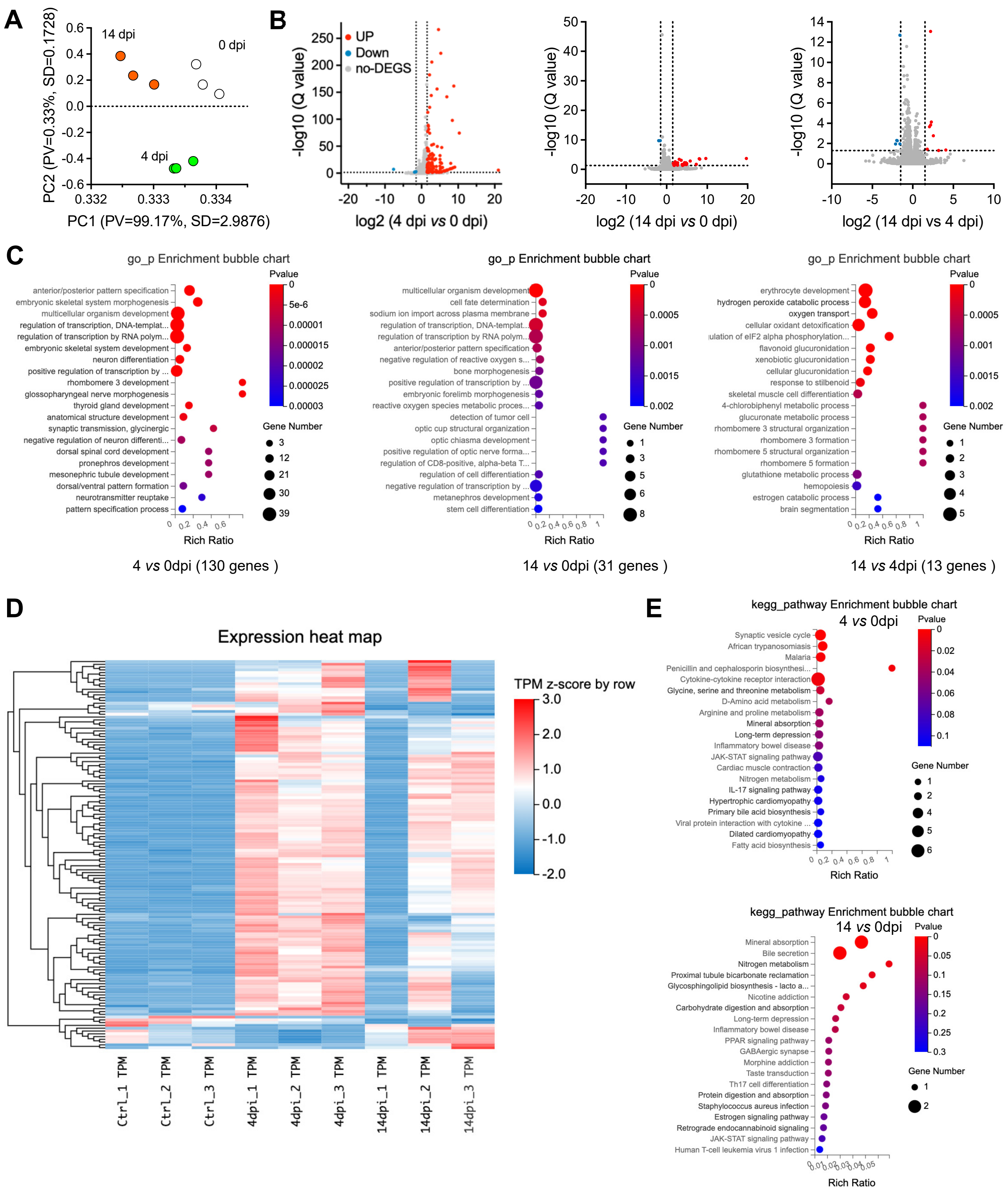

Figure S5. Transcriptomic profile overview in SARS2-N501Y$_{MA30}$ infected mouse brains. (A) Principal component analysis (PCA) of transcriptome profiles comparing mock-infected brains (0 dpi) with those at 4 and 14 dpi. **(B)** Volcano plot analysis: The x-axis denotes log2-transformed fold changes, and the y-axis indicates -log10-transformed significant values (Thresholds set at |log2fold| ≥ 1.5, Q value ≤ 0.05). **(C)** GO enrichment analysis of DEGs across groups. **(D)** Heatmap visualization of 140 DEGs identified post-infection. **(E)** KEGG pathway analysis comparing groups at 4 dpi vs 0 dpi, 14 dpi vs 0 dpi.

Supplemental Figure S6

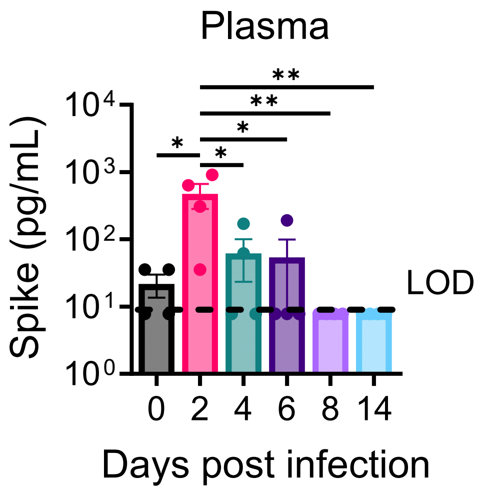

Figure S6. Detection of SARS-CoV-2 spike protein in plasma of infected mice by ELISA. C57BL/6 mice were intranasally infected with 10^4 PFU of SARS-CoV-2-N501Y$_{MA30}$. Plasma was collected at 0, 2, 4, 6, 8, and 14 dpi via submandibular blood collection. SARS-CoV-2 spike protein levels in plasma were determined using a SARS-CoV-2 spike RBD ELISA Kit. (n=4 per time point). Statistical significance: *$p<0.05$ and **$p<0.01$ were determined by ordinary one-way ANOVA. Data are presented as mean $\pm$ SEM.

Supplemental Figure S7

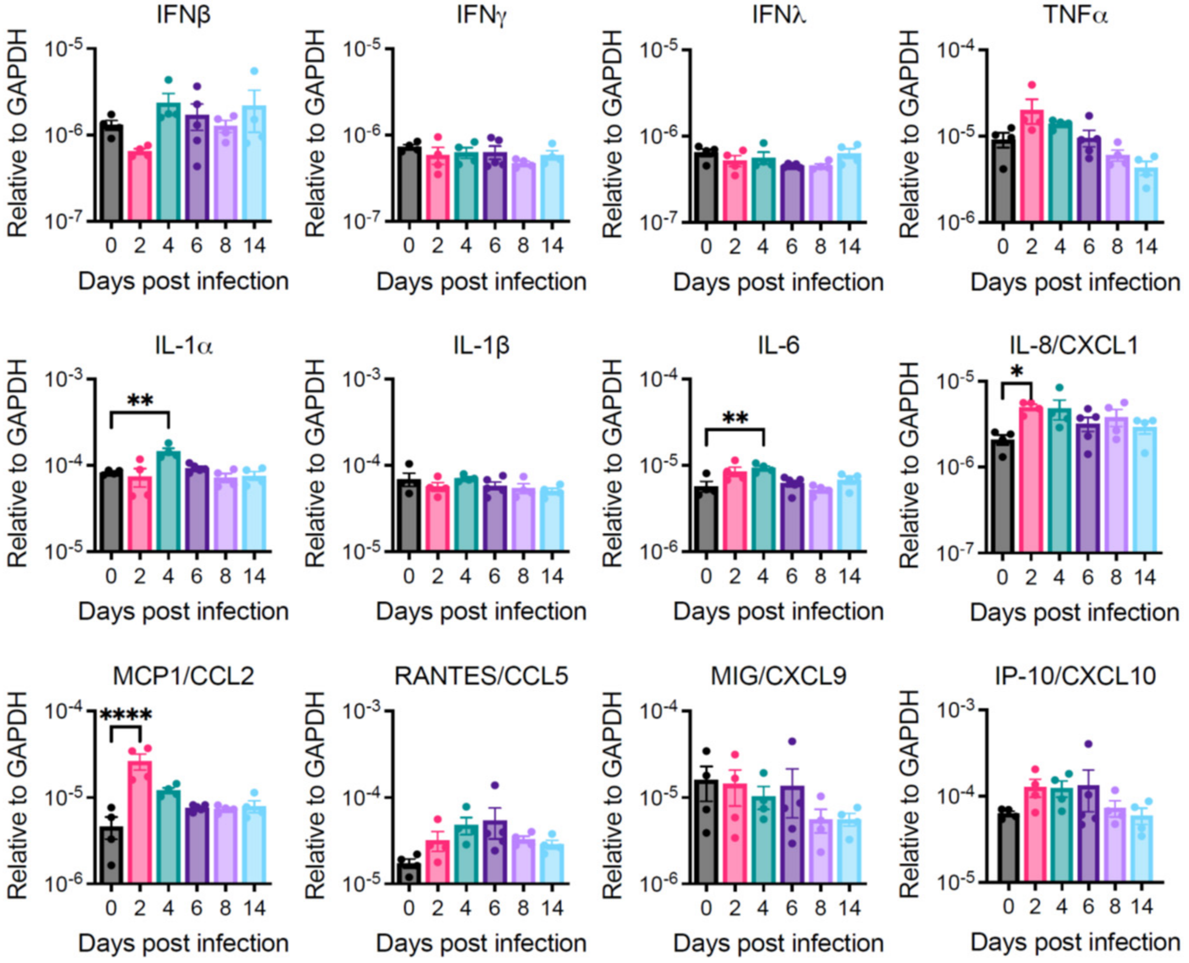

Figure S7. Cytokines and chemokines induced in the brain of SARS-CoV-2-infected mice. Brains of C57BL/6 mice intranasally infected with 10^4 PFU of SARS2-N501Y$_{MA30}$ were harvested at indicated dpi. Cytokine and chemokine transcripts were measured by qRT-PCR analyzing total RNA extracted from mock-infected (0 dpi) and infected young C57BL/6 mice. Each brain was collected from one individual mouse. Mock (0 dpi), 2, 4, 8, 14 dpi: n=4; 6 dpi: n=5. The levels of transcripts were normalized to GAPDH and presented as 2^-ΔCT. Statistical significance: *p<0.05, **p<0.01, and ****p<0.0001 were determined by ordinary one-way ANOVA. Data are presented as mean ± SEM.

Table S1

Gene	Forward Primer	Reverse Primer
2019-nCoV_N1	GACCCCAAAATCAGCGAAAT	TCTGGTTACTGCCAGTTGAATCTGG
SARS-CoV-2 sgRNA for E protein	CGATCTCTTGTAGATCTGTTCTC	ATATTGCAGCAGTACGCACACA
mouse GAPDH	AACAGCAACTCCCACTCTTC	CCTGTTGCTGTAGCCGTATT
mouse IFNα4	TCCATCAGCAGCTCAATGAC	AGGAAGAGAGGGCTCTCCAG
mouse IFNβ	TCAGAATGAGTGGTGGTTGC	GACCTTTCAAATGCAGTAGATTCA
mouse IFNγ	CGGCACAGTCATTGAAAGCCTA	GTTGCTGATGGCCTGATTGTC
mouse IFNλ	AGCTGCAGGTCCAAGAGCG	GGTGGTCAGGGCTGAGTCATT
mouse TNFα	GAACTGGCAGAAGAGGCACT	AGGGTCTGGGCCATAGAACT
mouse IL1α	CGCTTGAGTCGGCAAAGAAAT	ACAAACTGATCTGTGCAAGTCTC
mouse IL-1β	ACTGTTTCTAATGCCTTCCC	ATGGTTTCTTGTGACCCTGA
mouse IL-6	GAGGATACCACTCCCAACAGACC	AAGTGCATCATCGTTGTTCATACA
mouse IL-8	CACCTCAAGAACATCCAGAGCT	CAAGCAGAACTGAACTACCATCG
mouse CCL2	CTTCTGGGCCTGCTGTTCA	CCAGCCTACTCATTGGGATCA
mouse CCL5	AGATCTCTGCAGCTGCCCTCA	GGAGCACTTGCTGCTGGTGTAG
mouse CXCL2	CATCCAGAGCTTGAGTGTGACG	GGCTTCAGGGTCAAGGCAAACT
mouse CXCL9	GCCATGAAGTCCGCTGTTCT	GGGTTCCTCGAACTCCACACT
mouse CXCL10	GCCGTCATTTTCTGCCTCAT	GCTTCCCTATGGCCCTCATT
mouse Mx1	TGGACATTGCTACCACAGAGGC	TTGCCTTCAGCACCTCTGTCCA
mouse OAS1	ATTACCTCCTTCCCGACACC	CAAACTCCACCTCCTGATGC
